# Experimental Mis-Splicing Assessment and ACMG/AMP-Guided Classification of 47 *ATM* Splice-Site Variants

**DOI:** 10.3390/ijms27020765

**Published:** 2026-01-12

**Authors:** Inés Llinares-Burguet, Lara Sanoguera-Miralles, Elena Bueno-Martínez, Ada Esteban-Sanchez, Daniel Romano-Medina, Lobna Ramadane-Morchadi, Alicia García-Álvarez, Pedro Pérez-Segura, Doug F. Easton, Peter Devilee, Maaike P. G. Vreeswijk, Miguel de la Hoya, Eladio A. Velasco-Sampedro

**Affiliations:** 1 Splicing and Genetic Susceptibility to Cancer, Unidad de Excelencia Instituto de Biomedicina y Genética Molecular de Valladolid (IBGM), Consejo Superior de Investigaciones Científicas—Universidad de Valladolid (CSIC-UVa), 47003 Valladolid, Spain; ines.llinares@estudiantes.uva.es (I.L.-B.); lara.sanoguera@uva.es (L.S.-M.); elena.bueno@uva.es (E.B.-M.); dani.romanom@gmail.com (D.R.-M.); aliciaga@uva.es (A.G.-Á.); 2 Molecular Oncology Laboratory, Hospital Clínico San Carlos, IdISSC (Instituto de Investigación Sanitaria del Hospital Clínico San Carlos), 28040 Madrid, Spainlobna.ramadane@salud.madrid.org (L.R.-M.); pedro.perez@salud.madrid.org (P.P.-S.); 3 Centre for Cancer Genetic Epidemiology, Department of Public Health and Primary Care, University of Cambridge, Cambridge CB1 8RN, UK; dfe20@medschl.cam.ac.uk; 4 Department of Human Genetics, Leiden University Medical Center, 2333 ZA Leiden, The Netherlands

**Keywords:** *ATM*, ataxia–telangiectasia, hereditary breast cancer, splicing, aberrant splicing, VUS, splice-site variants, minigenes, ACMG/AMP

## Abstract

Pathogenic germline variants in the *ATM* gene are associated with a 20–30% lifetime risk of breast cancer. Crucially, a relevant fraction of loss-of-function variants in breast cancer susceptibility genes disrupts pre-mRNA splicing. We aimed to perform splicing analysis of *ATM* splice-site variants identified in the large-scale sequencing project BRIDGES (Breast Cancer After Diagnostic Gene Sequencing). To this end, we bioinformatically selected 47 splice-site variants across 17 exons that were genetically engineered into three minigenes and assayed in MCF-7 cells. Aberrant splicing was observed in 38 variants. Of these, 30 variants, including 7 missense, yielded no or negligible expression of the minigene full-length (mgFL) transcript. A total of 69 different transcripts were characterized, 48 of which harboured a premature termination codon. Some variants, such as c.2922-1G>A, generated complex patterns with up to 10 different transcripts. Alternative 3′ or 5′ splice-site usage was the predominant event. Integration of *ATM* minigene read-outs into the ACMG/AMP (American College of Medical Genetics and Genomics/Association for Molecular Pathology)-based specifications for the *ATM* gene enabled the classification of 30 *ATM* variants as pathogenic or likely pathogenic and 9 as likely benign. Overall, splicing assays provide key information for variant interpretation and the clinical management of patients.

## 1. Introduction

The ataxia–telangiectasia-mutated (*ATM*) gene (MIM# 607585) encodes a protein essential for maintaining genomic integrity, acting as a master regulator of the DNA damage response (DDR) through the phosphorylation of hundreds of downstream targets [1]. DDR dysregulation is known to cause multiple human genetic disorders [2], including chromosome instability disorders and an increased susceptibility to cancer [3]. Indeed, Ataxia–Telangiectasia (MIM#208900) is a rare inherited neurological disorder caused by bi-allelic loss-of-function (LOF) variants in *ATM* [4,5]. Heterozygous protein-truncating variants (PTVs) in *ATM* are associated with a roughly twofold increased risk of breast cancer (BC) and markedly elevated risks for other malignancies, including pancreatic cancer (~6.5-fold), gastric cancer (~3.0-fold), and prostate cancer (~2.6-fold) [6,7,8,9,10,11,12,13]. Moreover, *ATM* PTVs show a stronger association with estrogen receptor (ER)–positive breast cancer than with ER-negative breast cancer [10,11]. The National Comprehensive Cancer Network Guidelines (https://www.nccn.org/guidelines/guidelines-detail?category=2&id=1545, accessed on 12 May 2025) recommend annual mammography starting at age 40 and breast magnetic resonance imaging to be initiated between ages 30 and 35 for *ATM* pathogenic variant carriers. This strategy has been estimated to reduce BC mortality by more than 60% [14].

RNA splicing is a crucial and highly regulated step in gene expression, orchestrated by the spliceosome and controlled by a large array of recognition and regulatory sequences [15,16,17]. Splicing dysregulation induced by genetic variants at the 5′ and 3′ splice-sites (5′ss and 3′ss, respectively) is a well-established mechanism of pathogenicity [18,19,20,21]. Recent estimates suggest that approximately one-third of all disease-causing variants disrupt splicing [22]. This is exemplified by the *ATM* gene, where previous studies found that roughly half of pathogenic variants cause mis-splicing [8,23,24]. While variants at the ±1 and ±2 positions of the splice-sites are considered strong indicators of pathogenicity [25], predicting the splicing impact of variants at these and other splice-site positions remains challenging. Regardless of the specific nucleotide or motif affected, experimental splicing data are crucial for the clinical interpretation of variants. In this regard, splicing reporter minigenes represent valuable tools to test variants in any disease-responsible gene [26,27,28].

This work expands upon our previous splicing analyses of the breast cancer susceptibility genes *RAD51C*, *RAD51D*, *PALB2*, *ATM*, and *CHEK2*, conducted within the Breast Cancer After Diagnostic Gene Sequencing (BRIDGES) project (https://cordis.europa.eu/project/id/634935, accessed on 12 May 2025), where 34 known or suspected BC susceptibility genes were sequenced in 60,466 BC cases and 53,461 controls [10]. We conducted extensive splicing analyses of 259 variants in the five BC susceptibility genes described above using minigene assays [29,30,31,32,33]. A total of 124 were classified as (likely) pathogenic (LP/P) based on the guidelines of the American College of Medical Genetics and Genomics and the Association for Molecular Pathology (ACMG/AMP).

*ATM* is a very large gene comprising 63 exons, which complicates the splicing analysis of candidate variants distributed throughout its sequence. Building upon our initial investigation of 56 *ATM* splice-site BRIDGES variants across 22 exons [32], we have extended our analysis to another set of 47 preselected BRIDGES variants with a view to providing a more comprehensive functional evaluation of *ATM* splicing alterations. These variants were assessed using three new minigenes covering 17 additional exons. The resulting splicing data have ultimately contributed to a tentative clinical classification based on the ClinGen Hereditary Breast, Ovarian and Pancreatic Cancer Variant Curation Expert Panel (HBOP VCEP) Specifications for *ATM* variants [34].

## 2. Results

### 2.1. Wild Type Minigene Assays

**Figure 1:**
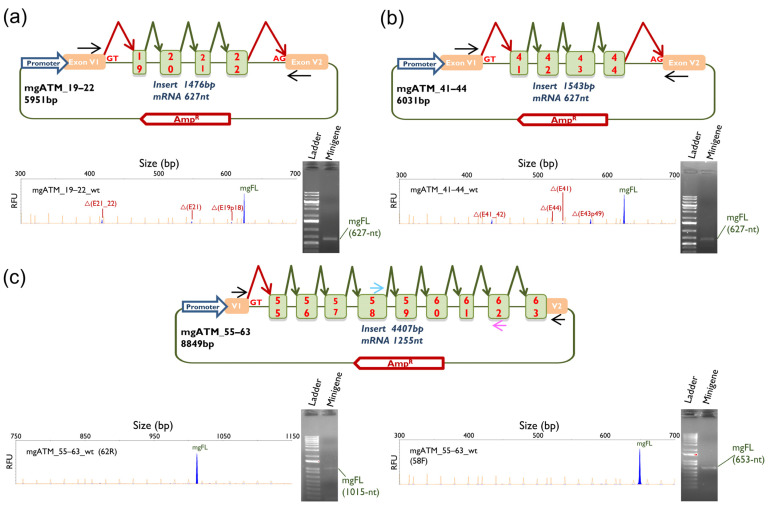
Schematic representation and splicing assay of the wt minigenes: (**a**) mgATM_19–22; (**b**) mgATM_41–44; (**c**) mgATM_55–63. Exons are indicated by boxes; green and red elbow arrows indicate the expected splicing reactions and black arrows locate specific vector RT-PCR primers. Blue and pink arrows in mgATM_55–63 locate primers RT-ATM-58F and RT-ATM-62R, respectively. RT-PCR products were analyzed by agarose gel electrophoresis (**right**) and fluorescent fragment electrophoresis (**left**). mgFL, expected minigene full-length transcript. Transcript descriptors: Δ, skipping of exonic sequences; E, exon; p, acceptor shift (the exact number of nucleotides is indicated). The *x*-axis indicates size in base pairs (bp) (electropherograms on the top), and the *y*-axis represents Relative Fluorescence Units (RFU).

The three *ATM* wild type (wt) minigenes (mgATM_19–22, mgATM_41–44 and mgATM_55–63) were functionally assayed in MCF-7 cells. All three constructs predominantly yielded the expected minigene full-length (mgFL) transcripts (83.5%, 76.2% and 100%, respectively). In addition, several naturally occurring alternative transcripts were identified: Δ(E19p18), Δ(E21) and Δ(E21_E22) in mgATM19–22; Δ(E41), Δ(E41_E42), Δ(E43p49) and Δ(E44) in mgATM_41–44 (Table 1 and Figure 1). These alternative splicing events have been reported in the reference 300K-RNA dataset, with SpliceVault detection rates ranging from 0.2% for Δ(E41) to 16.1% for Δ(E43p49) [35].

### 2.2. ATM Variants Assays

**Figure 2:**
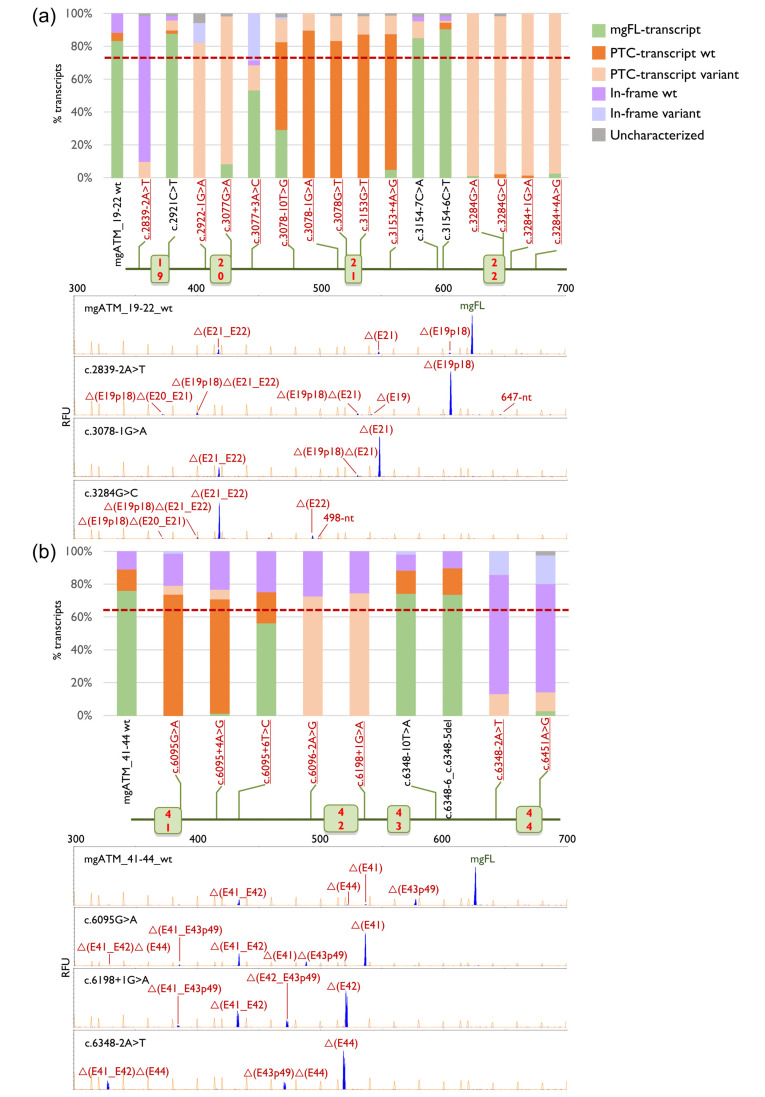

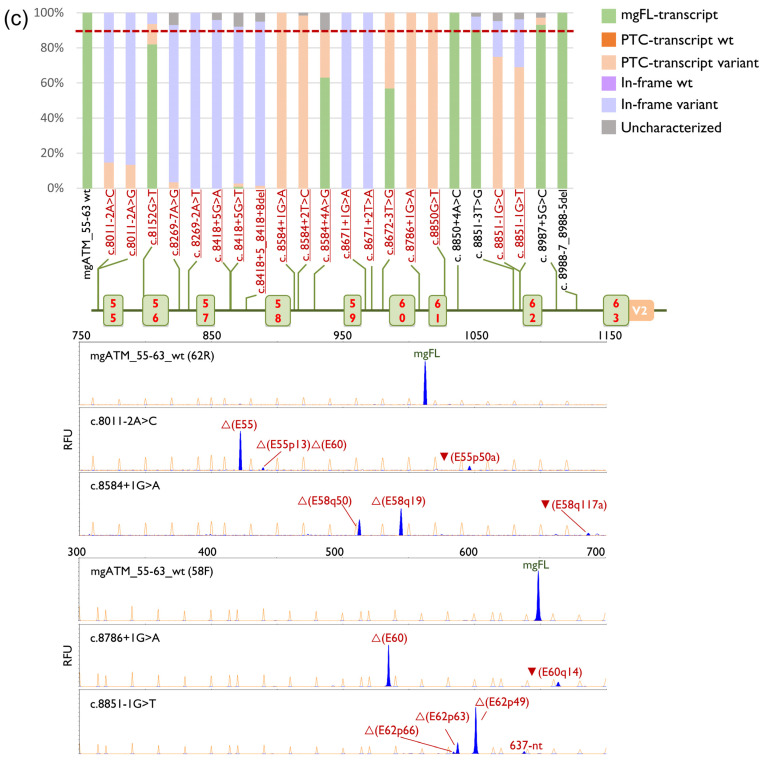
Splicing outcomes of selected *ATM* variants in minigenes: (**a**) mgATM_19–22; (**b**) mgATM_41–44; (**c**) mgATM_55–63. Graphical representation of the relative contribution of each type of transcript (**top**), map of tested variants (**middle**) and fluorescent fragment electrophoresis of wt and two representative variants (**bottom**). Spliceogenic variants are highlighted in red and underlined. Bar graph legend: The dashed red line indicates the threshold above which a variant is considered spliceogenic. PTC-transcript wt and In-frame wt, alternative transcripts produced by the wt minigene; PTC-transcript variant and In-frame variant, transcripts induced by the corresponding variants.

Wild type minigenes were used as template to genetically engineer the 47 candidate variants: 16 in mgATM_19–22, 9 in mgATM_41–44 and 22 in mgATM_55–63. Consistent with our previous report [32], a 10% reduction in the mgFL transcript was adopted as a conservative operational threshold for spliceogenic variants. This threshold serves to exclude weak impacts on splicing or inter-experimental variability that may result in minor fluctuations in transcript ratios. Analysis of results revealed that 38 out of 47 (81%) variants disrupted splicing, 30 of which exhibited a severe impact on splicing, resulting in neglible (<5%) or no detectable expression of mgFL-transcript (Table 1, Figure 2, Supplementary Figure S1). To check splicing reproducibility, two variants (c.3284G>A and c.3154-6C>T) and the wild type minigene mgATM_19–22 were also assayed in MDA-MD-231 cells, showing very similar outcomes (Supplementary Figure S2).

Seventeen spliceogenic variants affected the canonical ±1 and ±2 positions, each resulting in the production of 100% aberrant transcripts. The remaining 21 variants altered other exonic or intronic nucleotides within the splice-site consensus sequence, producing splicing defects ranging from weak–moderate perturbation to complete loss of normal splicing. (Table 1, Figure 2, Supplementary Figure S1). Interestingly, one exon 19 variant (c.2921C>T, p.(Ser974Phe)) and two intron 21 variants (c.3154-7C>A, c.3154-6C>T) improved splicing efficiency, producing higher levels of mgFL compared to the wild type, (Table 1 and Figure 2a). Additionally, seven predicted missense variants (c.3078G>T, p.(Trp1026cys); c.3153G>T, p.(Glu1051Asp); c.3284G>A, p.(arg1095lys); c.3284G>C p. (Arg1095Thr); c.6095G>A p.(Arg2032lys); c.6451A>G, p.(Arg2151Gly); c.8850G>T p.(Glu2950Asp)) also induced severe splicing anomalies. These variants resulted in very low or undetectable levels of mgFL-transcripts. Notably, the nonsense variant (c.3077G>A, p.(Trp1026*)) generated only 8.5% mgFL-transcripts. Forty-one variants generated two or more transcripts, which illustrate the complexity of variant-induced splicing outcomes.

A total of 69 different transcripts were characterized by fluorescent-fragment analysis, including five mgFL-transcript carrying exonic variants (r.2921c>u, r.3077g>a, r.3284g>a, r.6451a>g and r.8152g>u) (Supplementary Table S1). Remarkably, variant c.3077G>A, which introduces, a stop codon [p.(Trp1026*)], produced 90% of transcripts with premature termination codon (PTC) (Figure 2a and Supplementary Figure S2). Twelve transcripts could not be characterized (1–9.4% of the overall expression) (Table 1 and Figure 2). Fourteen transcripts preserved the reading frame, whereas 48 transcripts were predicted to introduce a PTC, including mgFL-c.3077G>A, of which 46 were predicted to elicit the Nonsense-Mediated Decay (NMD) (Figure 2, Supplementary Table S1). The predominant splicing event (30 transcripts) was alternative usage of cryptic 5′ss or 3′ss, which was observed in 28 variants. Particularly, transcript ▼(E56p182) used a cryptic 5′ss strengthened by the fusion of two segments of a shortened intron 56 (a reduction from 7260 bp of the original intron to 567 bp) in the minigene (MaxEntScan (MES) natural 1.04 → MES chimeric ivs 4.04). Single/multiexon skipping was identified in 19 transcripts, while exon skipping combined with alternative splice-site usage was concomitantly detected in 11 different transcripts. Finally, one transcript contained full retention of intron 62 [▼(I62)] (Supplementary Table S1).

### 2.3. ACMG-AMP-Based Classification of Variants

According to the ACMG/AMP-based specifications of the *ATM* gene, 26 variants were classified as pathogenic (P), 4 as likely pathogenic (LP), 9 as likely benign (LB), and 8 as Variants of Uncertain Significance (VUS) (Table 2, Supplementary Tables S2 and S3). It is worth mentioning that seven predicted missense variants were first characterized as spliceogenic and subsequently classified as P. Of the 47 variants under investigation, 35 had been reported previously in ClinVar (https://www.ncbi.nlm.nih.gov/clinvar/?term=atm%5Bgene%5D&redir=gene; last accessed on 23 October 2025) with classification confidences ranging from conflicting/single submitter (*N* = 8) to expert panel review (*N* = 4) (Table 2, Supplementary Table S3). Our minigene-based classification showed full concordance with expert panel review. Specifically, it reclassified three out of eight VUSs as P (*n* = 1), LP (*n* = 1), or LB (*n* = 1). Furthermore, three conflicting variants (VUS/LB) were catalogued as LB, while one conflicting variant (LP/VUS) was upgraded to P. Further, the minigene-based classification reached a definite pathogenic status (≥ +10 points) for most variants reported as P/LP (11 out of 13).

## 3. Discussion

Massively parallel sequencing of breast and ovarian cancer susceptibility genes is a highly sensitive, cost-effective, and rapid approach for detecting risk-associated variants, ultimately improving the clinical management of patients [10,11,36,37]. Despite these advancements, a significant limitation is the frequent detection of VUS [38]. Accurately classifying these VUS poses a challenge for genetic counselling. Since splicing dysregulation is a prevalent etiopathogenic mechanism in BC susceptibility genes [39], the assessment of VUS via splicing studies is essential for proper classification and improved patient management.

Several in silico tools, such as MES or SpliceAI, predict variant impact with a certain degree of accuracy. However, functional studies are still necessary to verify these predictions. Key parameters, such as the level of splicing alteration for variants that do not affect the AG/GT dinucleotides or the number and type of transcripts generated by each variant, are beyond the current capabilities of these tools. For these reasons, experimental validation is key for assessing the potential pathogenicity of predicted splice-altering variants. When RNA from carriers is not available, hybrid minigenes are an effective tool for evaluating potential spliceogenic variants in virtually any human disease gene [40,41,42,43,44]. So far, we have built seven *ATM* minigenes: mgATM_4–9, mgATM_11–17, mgATM_19–22, mgATM_25–29, mgATM_41–44, mgATM_49–52 and mgATM_55–63. These clones cover 39 of the 63 *ATM* exons (62% of the coding sequence). According to the ClinVar database, 11500 *ATM* reported variants are located within the regions covered by our minigenes, illustrating their potential for large-scale splicing studies.

As artificial constructs, minigenes have inherent limitations. These include the shortening of long introns that may harbour internal regulatory sequences, potentially impacting splicing events. Furthermore, while minigene assays rely on a single insert with a specific haplotype (typically the reference sequence), the genetic background of each individual remains a critical factor, given that SNPs can modulate alternative splicing [45]. On the other hand, they also offer a number of advantages that we have previously underscored in other studies [46,47]. These can be summarized as: (1) analysis of a single mutant allele without the interference of the wild type allele; (2) use of disease-relevant cell lines; (3) NMD inhibition that improves the detection of all PTC-NMD transcripts; (4) high sensitivity and resolution of fluorescent capillary electrophoresis for the detection of transcripts; (5) high reproducibility of variant-induced outcomes [30,32]. Notably, the minigene results of eight variants from this study were supported by previous RNA studies in carriers (Supplementary Table S4): c.2922-1G>A (c.2922-2A>G carrier: Δ(E20p32) vs. Δ(E20p32) and Δ(E20p71) as main minigene transcripts) [38], c.3078-1G>A (Δ(E21)) [39], c.6095G>A (Δ(E41) in four independent studies) [17,40,41,42], c.6198+1G>A (Δ(E42) vs. Δ(E42) and Δ(E41_E42) as main minigene transcripts) [48], c.8418+5_8418+8del (Δ(E57)) [43], c.8418+5G>A (Δ(E57)) [44], c.8584+2T>C (Δ(E58q19)) [45], c.8786+1G>A (Δ(E60) and ▼(E60q14)) [46,47] and c.8851-1G>T (Δ(E62p63) vs. Δ(E62p49) and Δ(E62p63) in minigenes) [48]. The reliability of the minigene analysis was further supported by the strong concordance observed with SpliceAI-visual predictions [49] and SpliceVault top events [35], across the evaluated cases (Supplementary Table S4). The single exception was c.6095+6T>C for which SpliceAI predicted no spliceogenic effect, while mgATM_41–44 revealed a leaky splicing variant producing substantial levels of Δ(E41_E42) transcripts. This finding, together with the fact that SpliceAI-visual does not predict multi-exon skipping -as observed for exon 22 donor site, exon 42 acceptor site, and exon 42 donor site variants- underscores the critical relevance of experimental splicing assays to complement in silico predictions.

We formerly carried out exhaustive studies of 56 *ATM* splice-site variants and 62 *ATM* SRE (Splicing Regulatory Element)-variants by minigene assays [32,47,50]. Here, 47 new *ATM* splice-site variants were tested using three different minigenes with 17 exons, which were designed to closely replicate the genomic context of the *ATM* gene. The selection strategy for splice site variants was successful in our two studies of the *ATM* gene [32], with 84% of the selected variants disrupting splicing. Most spliceogenic variants of the present study (21/38, 55%) involved changes at conserved positions of the 5′ss and 3′ss beyond the canonical ±1,2 dinucleotides, demonstrating that any DNA change can be deleterious and may contribute to cancer risk. In addition to the intronic changes, seven missense variants severely disrupted splicing with no traces or negligible amounts of mgFL-transcripts (Table 1). Specifically, these variants primarily affected the last exon nucleotide (5 variants), but also the first and penultimate nucleotides.

Minigene analysis further revealed the high complexity of *ATM* splicing patterns, with a total of 69 different transcripts detected. This intricate landscape poses a challenge for clinical interpretation, particularly for variants like c.2922-1G>A, which generated up to 10 distinct transcripts.

### Clinical Interpretation of Variants

Overall, minigene data made a major contribution to the classification of most variants, as illustrated in Table 2. In keeping with that, the minigene readouts for four variants were considered inconclusive (i.e., not contributing to the final classification) and were ultimately classified as VUS. In all four cases, minigene inconclusiveness was caused by the evidence of a partial effect on splicing (i.e., leaky variants). Interestingly, two of these four leaky variants have been assessed in a recent MAVE (Multiplexed Assays of Variant Effect) study [51] displaying different results. The c.8672-3T>G variant (with full-length transcripts contributing 57.1% of the expression in our minigene study) scored non-functional. By contrast, the c.8584+4A>G variant (full-length transcripts contributing 63.4% of the expression in our minigene study), scored as intermediate function. In both cases, MAVE data was reported with high confidence. Assuming that the leakiness level observed in our minigene assay mirrors that of the MAVE model (HCT116 cell line), the data supports a dosage sensitivity model in which *ATM* variant alleles expressing as much as 57% of full-length transcripts are fully pathogenic. These results sharply contrast with a previously proposed dosage-sensitive expression model that considered *ATM* alleles producing >30% full-length transcripts to be benign [32]. Such discrepancies underscore the challenge of developing robust dosage-sensitive expression models and reinforce the need for extreme caution when assigning the PVS1_(RNA) or BP7_(RNA) evidence to leaky variants.

In the future, robust dosage-sensitive expression models for *ATM* (or for other clinically relevant genes) could be developed by grouping variants with similar degrees of ‘leakiness’ for case–control burden analyses. Moreover, MAVEs that assess protein function and splicing profiles simultaneously should further improve model accuracy.

Among the 47 variants investigated, 17 affected canonical acceptor or donor positions (±1,2) and therefore carried a predictive PVS1 code of variable strength assigned by the HBOPC VCEP (Supplementary Table S2). In most cases, PVS1_(RNA) evidence from minigene readouts confirmed the VCEP predictions. The single discordant case was c.8269-2A>T. The VCEP recommended PVS1 at supporting strength for this exon 57 acceptor-site change because the conservative prediction favoured the short in-frame acceptor shift Δ(E57p18). Our minigene data, however, revealed that complete exon 57 skipping Δ(E57) contributed >70% of the overall signal, supporting PVS1_(RNA) at full strength.

Of note, variant c.2839-2A>T, reported as Pathogenic in ClinVar by a single submitter, was classified as VUS in our study. This conservative classification is supported by minigene data demonstrating an acceptor shift Δ(E19p18) caused by the activation of an exonic cryptic acceptor site. This acceptor shift results in an in-frame deletion, p.(Tyr947_Lys952del), which, when assessed against the *ATM*-specific PVS1 decision tree, provides only supporting evidence for pathogenicity. The absence of a functional score for c.2839-2A>T in the *ATM* MAVE study coupled with benign-supporting scores reported for closely related substitutions at the same site further support our conservative assessment [51].

In summary, we have carried out the most comprehensive splicing studies of the *ATM* gene to date, testing a total 165 variants (103 splice-site and 62 SRE variants) by minigene assays [32,47,50]. Our results demonstrate that a significant proportion (60%) of these variants impair splicing, with 42% being classified as (likely) pathogenic based on ACMG/AMP-based specifications refined for *ATM*. Future burden analyses using case–control or family data will be essential to further refine the classification method, and to provide accurate risk estimations that inform the clinical management of BC patients.

From a therapeutic perspective, glucocorticoid treatment has shown promising results in Ataxia–Telangiectasia patients by alleviating neurological symptoms. This effect is mediated by the generation of shorter *ATM* transcripts (specifically exons 3–52, 4–53, and 2–52), which encode a (partially) functional protein termed mini-ATM [52]. P/LP variants associated with splicing defects in exons 19–22 and 41–44 would theoretically result in ‘mini-ATM’ transcripts (exons 3–52, 4–53, and 2–52) that still retain the underlying splicing anomalies. Consequently, it remains uncertain whether these specific isoforms could provide even a partial rescue of ATM function. Conversely, Ataxia–Telangiectasia patients carrying P/LP variants within exons 55–63, which are nor part of the mini-ATM structure, may represent a cohort that could benefit from glucocorticoid therapy.

Collectively, our findings provide compelling evidence that variant-induced splicing disruptions are a major disease mechanism for *ATM* and, more broadly, for other BC susceptibility genes [29,31,32,33,53,54,55,56]. The ClinGen specifications for the *ATM* gene offer a valuable framework for the clinical interpretation of variants when splicing data are available. This work provides a strong rationale for systematically incorporating splicing data, such as that generated by minigene assays, into variant classification pipelines and genetic diagnostics to enhance patient care.

## 4. Materials and Methods

Ethical approval for this study was obtained from the Ethics Committee of the Spanish National Research Council-CSIC (28 May 2018).

### 4.1. Annotation and Selection of Candidate Variants

Variants, transcripts and predicted protein products were annotated following the Human Genome Variation Society guidelines (HGVS: https://hgvs-nomenclature.org/stable/, accessed on 12 May 2025), using the MANE selected transcript of *ATM* (GenBank NM_000051.4; Ensembl ENST00000675843.1). To simplify, generated transcripts were described with abbreviated annotations as previously described [57].

We previously selected potentially spliceogenic variants at the intron-exon boundaries based on: (i) ≥20% decrease in MaxEntScan (MES) scores [58,59,60]; (ii) creation of putative de novo sites (MES cutoff ≥ 3.0); or (iii) changes at conserved positions (−3, −2, −1, exon 5′-3′-ends, +3, +4, +5, and +6) of the consensus splice-sites, regardless of MES predictions [33]. Of the 381 variants at the *ATM* exon/intron boundaries originally identified in the BRIDGES project, 136 had been previously selected, 56 of which had been tested [32]. Here, we focus on another 47 candidate variants located at the *ATM* exons 19–22, 41–44 and 55–63.

### 4.2. Minigenes Construction and Site-Directed Mutagenesis

Three inserts with the target exons and their flanking intronic sequences were designed in-house to obtain the minigenes mgATM_19–22, mgATM_41–44 and mgATM_55–63 (Figure 1; Supplementary Figure S3; Supplementary Table S5).

Minigene mgATM_19–22 (5951 bp) was created by deleting exons 17–18 from mgATM_17–22, which had not yielded a clean splicing pattern in a previous study [32]. The mgATM_41–44 construct (6031 bp) was assembled in three consecutive steps using overlap extension PCR [61], as previously described [62]. Similarly, minigene mgATM_55–63 was assembled in two steps. First, the insert with exons 55–63 (4407 bp) was synthesized (GeneArt, Thermo Fisher Scientific, Waltham, MA, USA) and cloned into the splicing plasmid pSAD between the *EagI* and *SalI* restriction sites. Then, the last 88 nt of *ATM* exon 63 and 455 nt of vector intron were deleted to generate a chimeric exon 63-V2, obtaining the final minigene mgATM_55–63 (8307 bp) (Figure 1c; Supplementary Figure S3c; Supplementary Table S5). The final constructions were confirmed by sequencing (Macrogen, Madrid, Spain) and functionally assayed in MCF-7 cells (estrogen receptor-positive human breast adenocarcinoma cell line; ATCC HTB-22 [63]; LGC Standards, Barcelona, Spain) [64,65]. Selected variants were introduced into the wt minigene by site-directed mutagenesis (QuikChange Lightning kit, Agilent, Santa Clara, CA, USA) (Supplementary Table S5). Mutant minigenes were confirmed by sequencing (Macrogen).

### 4.3. Splicing Assays

Around 2 × 10^5^ MCF-7 cells were transiently transfected with 1 µg of wt or mutant minigenes. Transfection, NMD inhibition with cycloheximide, RNA purification and retrotranscription were performed as previously described [50]. To assess the reproducibility of the splicing outcomes, several variants were also tested in MDA-MD-231 cells.

**Figure 3:**
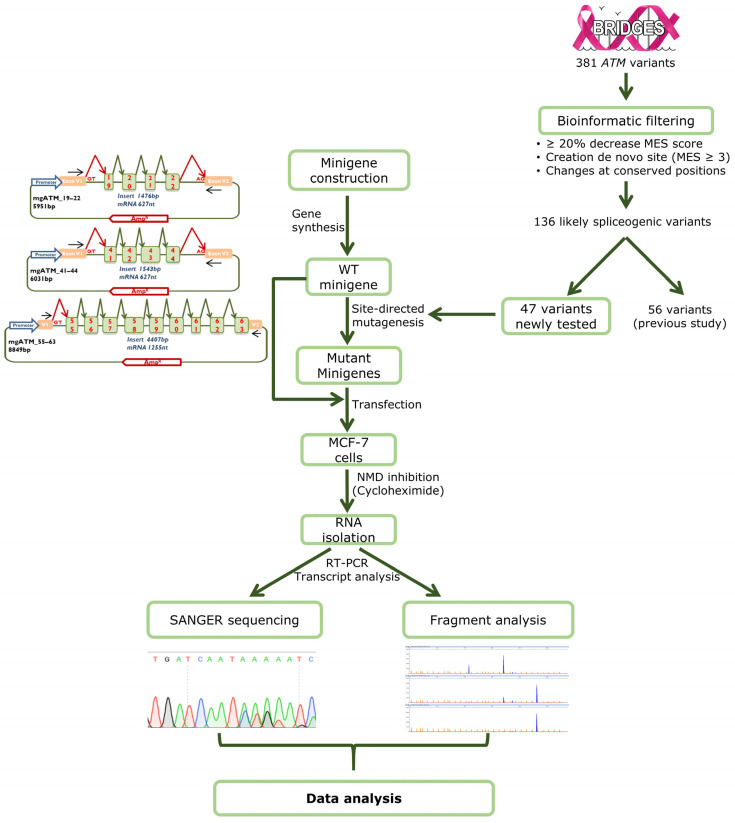
Workflow of the minigene strategy. The basic assay includes the following steps: (1) Minigene construction; (2) Bioinformatic filtering of variants; (3) Site-directed mutagenesis; (4) Transfection of the wild type and mutant minigenes; (5) Inhibition of Nonsense-mediated decay and RNA purification; (6) Transcript sequencing and fragment analysis by fluorescent capillary electrophoresis; (7) Data interpretation.

Forty ng of cDNA were amplified with Platinum Taq polymerase (Life Technologies, Carlsbad, CA, USA) as indicated in Supplementary Figure S4. To calculate the relative expression of each transcript, semiquantitative fluorescent RT-PCRs were performed in triplicate under the same conditions as indicated in Supplementary Figure S4, except that 26 cycles were applied. FAM-labelled PCR products were run with LIZ-1200 Size Standard (Life Technologies, Carlsbad, CA, USA) at the Macrogen facility and analyzed with the Peak Scanner software V1.0 (Life Technologies). Only peak heights > 50 RFU were considered to calculate the average relative proportions of each transcript and the corresponding standard deviations. Transcripts with a relative expression level below 1% were filtered out before downstream analysis. The complete workflow of minigene assays is shown in Figure 3.

### 4.4. ACMG/AMP-Based Tentative Classification of ATM Genetic Variants

*ATM* variants were classified according to an ACMG/AMP point system, a Bayesian framework that allows for increased flexibility and accuracy in combining different ACMG/AMP criteria and strengths of evidence [63,66]. In this framework, point-based variant classification categories are defined as follows: Pathogenic (P) ≥ +10; Likely Pathogenic (LP) +6 to +9; Variant of Uncertain Significance (VUS) 0 to +5; Likely Benign (LB) −1 to −6; and Benign (B) ≤ −7. Points were assigned to *ATM* variants following the HBOP VCEP Guidelines for *ATM* (ATM version 1.4.0) [34].

To assign a PVS1_Variable (RNA)/BP7_Variable (RNA) evidence strength to mgATM read-outs, each transcript was evaluated against the *ATM*-specific PVS1 decision tree developed by the HBOP VCEP [34]. For complex mgATM readouts involving two or more transcripts, we followed ClinGen Sequence Variation Interpretation Splicing Subgroup recommendations [67]. Accordingly, each transcript was evaluated against the *ATM*-specific PVS1 decision tree, and a final pathogenic or benign evidence strength was assigned based on the relative contributions of pathogenic and benign-supporting transcripts to total minigene expression. The full methodological details are reported elsewhere [32]. For most read-outs, either pathogenic or benign supporting signal accounted for more than 80% of the total signal, making the final assignment of PVS1_RNA or BP7_RNA straightforward. Four variants (c.3078-10T>G, c.6095+6T>C, c.8584+4A>G, and c.8672-3T>G) did not exhibit a predominant signal, as both pathogenic and benign evidence accounted for less than 70% of the total signal. Consequently, the minigene readouts were deemed inconclusive, and neither PVS1_(RNA) nor BPT_(RNA) classifications were assigned (see Supplementary Table S2 for further details).

Variant classification combined the PVS1_Variable (RNA)/BP7_Variable (RNA) evidence derived from minigene readouts with other applicable ACMG/AMP criteria. SpliceAI-based PP3/BP4 evidence was not used in the final classification because it is not applicable alongside PVS1_Variable[RNA] or BP7_Variable[RNA]. REVEL (Rare Exome Variant Ensembl Learner)-based PP3/BP4 was applied for full-length transcripts encoding missense variants. REVEL results were integrated into the classification of complex minigene readouts and contributed to calls as PVS1_S[RNA] or BP7_S[RNA] (Supplementary Table S2).

Functional pathogenicity or benignity scores (PS3_Variable/BS3_Variable) were derived from a recent MAVE study on *ATM* [51] that was considered to match the HBOP VCEP PS3_Variable/BS3_Variable rubric for *ATM* (failure-to-rescue studies in *ATM* null cell lines). Variants were assigned functional evidence points by the study’s reported score confidence: ±2 points for high confidence, ±1 point for medium-high confidence, and 0 points for medium confidence. Functional evidence was not applied to variants with intermediate functional scores or to scores annotated only with computational confidence (Supplementary Table S2). PS4 was not applied because BRIDGES case–control data showed no statistically significant, variant-level association for any of the variants under review. The HBOPC VCEP does not endorse the following codes for *ATM* and they were therefore excluded from evaluation: PS2, PM1, PM6, PP2, PP4, PP5, BS2, BS4, BP1, BP3, BP5, BP6. The overall classification process is summarized in Supplementary Tables S2 and S3. This ACMG/AMP–based classification does not constitute a ClinGen-endorsed final clinical interpretation for any variant. On the contrary, it is intended only to illustrate the challenges of incorporating complex minigene readouts into an ACMG/AMP classification framework.

## Figures and Tables

**Table 1:** Splicing outcomes of 47 *ATM* variants.

Variant	Bioinformatics Summary	Transcripts ^1^			
		mgFL	PTC ^2^	In-Frame	Uncharacterized
mgATM_19–22_WT		83.5% ± 0.4%	Δ(E21) [5% ± 0.2]	[Δ(E21_E22)] [8.9% ± 0.2%] Δ(E19p18) [2.6% ± 0.1%]	
**c.2839-2A>T**	3′ss (8.1 → −0.3)	-	[Δ(E19p18) Δ(E21)] [3% ± 0.1%] [Δ(E19p18) Δ(E20_E21)] [1.5% ± 0.1%] Δ(E19) [1.3% ± 0.1%] Δ(E19p18) Δ(E21_E22)] [4.1% ± 0.3%]	Δ(E19p18) [88.9% ± 0.4%]	647-nt [1.2% ± 0.1%]
**c.2921C>T**	5′ss (6.3 → 4.4)	88% ± 0.9%	Δ(E21) [1.9% ± 0.1%] [Δ(E19p18) Δ(E20_E21)] [1.8% ± 0.9%] [Δ(E21_E22)] [4.3% ± 0.3%]	Δ(E19p18) [2.8% ± 0.1%]	665-nt [1.2% ± 0.1%]
**c.2922-1G>A**	3′ss (8.6 → −0.2)	-	Δ(E20p32) [41.3% ± 1.3%] Δ(E20p71) [32.5% ± 0.4%] [Δ(E19_E20)] [3.2% ± 0.2%] [Δ(E19p18) Δ(E20p32)] [2.3% ± 0.1%] [Δ(E20_E21)] [1.4% ± 0.1%] [Δ(E19p18) Δ(E20_E21)] [1.7% ± 0.3%]	Δ(E20) [11% ± 0.5%] [Δ(E19p18) Δ(E20)] [1.1%]	535-nt [3.6%] 546-nt [1.9% ± 0.7%]
**c.3077G>A**	5′ss (7 → 2.2)	8.5% ± 0.7%	▼(E20q4a) ^1^ [84.8% ± 0.9%] [Δ(E20_E21)] [1.2%] Δ(E20q17) [2.6% ± 0.2%] [Δ(E21_E22)] [1.4% ± 0.1%]		552-nt [1.5% ± 0.1%]
**c.3077+3A>C**	5′ss (7 → 0.8)	53.4% ± 0.1%	▼(E20q4b) ^1^ [6.5% ± 0.5%] [Δ(E19_E20)] [5.4% ± 0.1%] [Δ(E19p18) Δ(E20_E21)] [2.2% ± 0.1%] [Δ(E20_E21)] [1.2%]	Δ(E20) [26.6%] Δ(E19p18) [2.9%] [Δ(E19p18) Δ(E20)] [1.8%]	
**c.3078-10T>G**	5′ss (4.4 → 3.1)	29.4% ± 1%	Δ(E21) [53.4% ± 0.5%] [Δ(E19p18) Δ(E21)] [2.1% ± 0.1%] [Δ(E19p18) Δ(E20_E21)] [1.6% ± 0.4%] [Δ(E21_E22)] [10.3% ± 0.5%]	Δ(E19p18) [1.2% ± 0.1%]	552-nt [1% ± 0.7%] 588-nt [1%]
**c.3078-1G>A**	3′ss (4.4 → −4.3)	-	Δ(E21) [89.8% ± 0.2%] [Δ(E19p18) Δ(E21)] [2.7% ± 0.1%] [Δ(E21_E22)] [7.5% ± 0.1%]		
**c.3078G>T**	3′ss (4.4 → 2.9)	-	Δ(E21) [83.5% ± 0.3%] [Δ(E19p18) Δ(E21)] [2.8%] [Δ(E19p18) Δ(E20_E21)] [1.3% ± 0.1%] [Δ(E21_E22)] [11.1% ± 0.3%]		588-nt [1.3%]
**c.3153G>T**	5′ss (10.0 → 7.8)	-	Δ(E21) [87.4% ± 1%] [Δ(E19p18) Δ(E21)] [2.7% ± 0.1%] [Δ(E19p18) Δ(E20_E21)] [1.3% ± 0.2%] ▼(E21q4) [1.3% ± 1.3] [Δ(E21_E22)] [5.9% ± 0.1%]		588-nt [1.4%]
**c.3153+4A>G**	5′ss (10 → 7.1)	5.1% ± 0.4%	Δ(E21) [82.5% ± 0.9%] [Δ(E19p18) Δ(E21)] [2.7% ± 0.1%] [Δ(E19p18) Δ(E20_E21)] [1% ± 0.1%] [Δ(E21_E22)] [7.7% ± 0.3%]		588-nt [1% ± 0.1%]
**c.3154-7C>A**	3′ss (8.3 → 6.3)	85.2% ± 0.4%	[Δ(E19p18) Δ(E20_E21)] [1.4% ± 0.2%] [Δ(E21_E22)] [8.9% ± 0.3%]	Δ(E19p18) [2.9%]	665-nt [1.6% ± 0.1%]
**c.3154-6C>T**	3′ss (8.3 → 6.3)	90.6% ± 2.1%	Δ(E21) [4.1% ± 1.8%] [Δ(E19p18) Δ(E20_E21)] [1.1% ± 0.5%]	Δ(E19p18) [2.7% ± 0.1%]	665-nt [1.5% ± 0.1%]
**c.3284G>A**	5′ss (7.6 → −0.6)	1.4%	Δ(E22) [8.2% ± 0.2%] [Δ(E19p18) Δ(E20_E21)] [2.2% ± 0.1%] [Δ(E21_E22)] [84.7% ± 0.3%] [Δ(E19p18) Δ(E21_E22)] [3.5% ± 0.1%]		
**c.3284G>C**	5′ss (7.6 → 0.8)	-	Δ(E22) [8.4% ± 0.1%] [Δ(E21_E22)] [85.2%] [Δ(E19p18) Δ(E20_E21)] [1.4%] [Δ(E19p18) Δ(E21_E22)] [3.4% ± 0.1%]		498-nt [1.6% ± 0.1%]
**c.3284+1G>A**	5′ss (7.6 → −0.6)	-	Δ(E22) [12.6% ± 0.2%] [Δ(E21_E22)] [82.9% ± 0.2%] [Δ(E19p18) Δ(E20_E21)] [1%] [Δ(E19p18) Δ(E21_E22)] [3.5% ± 0.1%]		
**c.3284+4A>G**	5′ss (7.6 → 3.11)	2.8% ± 0.1%	Δ(E22) [3.7% ± 0.2%] [Δ(E21_E22)] [89.2% ± 0.4%] [Δ(E19p18) Δ(E21_E22)] [4.3% ± 0.1%]		
mgATM_41–44_WT		76.2% ± 0.1%	Δ(E43p49) [11.4%] Δ(E41) [1.6% ± 0.1%]	[Δ(E41_E42)] [9.8% ± 0.2%] Δ(E44) [1%]	
**c.6095G>A**	5′ss (6.6 → 1.5)	-	Δ(E41) [73.9% ± 0.5%] [Δ(E41_E43p49)] [1.9% ± 0.2%] [Δ(E41) Δ(E43p49)] [3.5% ± 0.1%]	[Δ(E41_E42)] [19.5% ± 0.5%] [Δ(E41_E42) Δ(E44)] [1.2% ± 0.3%]	
**c.6095+4A>G**	5′ss (6.6 → 4)	1.4% ± 0.3%	Δ(E41) [69.6% ± 1.7%] [Δ(E41_E43p49)] [2.3% ± 0.3%] [Δ(E41) Δ(E43p49)] [3.6% ± 0.3%]	[Δ(E41_E42)] [23.1% ± 1.5%]	
**c.6095+6T>C**	5′ss (6.6 → 6)	56.4% ± 0.1%	Δ(E43p49) [7.4% ± 0.7%] Δ(E41) [11.5% ± 0.5%]	[Δ(E41_E42)] [24.7% ± 0.6%]	
**c.6096-2A>G**	3′ss (8.4 → 0.5)	-	Δ(E42) [60.1% ± 0.5%] [Δ(E42_E43p49)] [9.8% ± 0.2%] [Δ(E41_E43p49)] [2.9% ± 0.1]	[Δ(E41_E42)] [27.2% ± 0.3%]	
**c.6198+1G>A**	5′ss (7.8 → −0.4)	-	Δ(E42) [60.7% ± 0.3%] [Δ(E42_E43p49)] [11% ± 0.3%] [Δ(E41_E43p49)] [3% ± 0.1%]	[Δ(E41_E42)] [25.3% ± 0.1%]	
**c.6348-10T>A**	3′ss (7.9 → 5.3)	74.4% ± 0.7%	Δ(E43p49) [12.5% ± 1.2%] Δ(E41) [1.6% ± 0.2%]	[Δ(E41_E42)] [7% ± 0.3%] Δ(E44) [2.8% ± 0.1] [Δ(E41_E42) Δ(E44)] [1.7% ± 0.1%]	
**c.6348-6_6348-5del**	3′ss (7.9 → 6)	73.8% ± 0.4%	Δ(E43p49) [13.8% ± 0.1%] Δ(E41) [2.3%]	[Δ(E41_E42)] [8.6% ± 0.4%] Δ(E44) [1.5% ± 0.1%]	
**c.6348-2A>T**	3′ss (7.9 → −0.4)	-	[Δ(E43p49) Δ(E44)] [13.3% ± 0.2%]	Δ(E44) [72.6% ± 0.3%] [Δ(E41_E42) Δ(E44)] [14.1% ± 0.4%]	
**c.6451A>G**	5′ss (7.2 → 3.8)	2.9% ± 0.3%	[Δ(E43p49) Δ(E44)] [11.6% ± 0.1%]	[Δ(E41_E42)] [3.2% ± 0.8%] Δ(E44) [64.4% ± 0.7%] [Δ(E41_E42) Δ(E44)] [17.9% ± 0.2%]	
mgATM_55–63_WT (F+62R)		100%			
**c.8011-2A>C**	3′ss (7.1 → −0.9)	-	[Δ(E55p13) Δ(E60)] [6.3% ± 0.4%] ▼(E55p50a) [8.7% ± 0.4%]	Δ(E55) [85% ± 0.4%]	
**c.8011-2A>G**	3′ss (7.1 → −0.8)	-	[Δ(E55p13) Δ(E60)] [6.9% ± 0.2%] ▼(E55p50b) [6.7% ± 0.9%]	Δ(E55) [86.4% ± 0.9%]	
**c.8152G>T**	3′ss (5 → 2.7)	82.3% ± 1.2%	▼(E56p182) [11.7% ± 0.4%]	Δ(E56p39) [6% ± 0.8%]	
**c.8269-7A>G**	3′ss (6.7 → 1.9)	-	▼(E57p139) [3.7% ± 0.2%]	Δ(E57) [8.5% ± 0.4%] ▼(E57p6) [81.1% ± 0.5%]	1193-nt [6.7% ± 0.4%]
**c.8269-2A>T**	3′ss (6.7 → −1.7)	-		Δ(E57) [72.5% ± 0.9%] Δ(E57p18) [27.5% ± 0.9%]	
**c.8418+5G>A**	5′ss (10.1 → 3.5)	-	▼(E57q23a) ^3^ [1.6%]	Δ(E57) [93.6% ± 0.1%]	1105-nt [4.8% ± 0.1%]
**c.8418+5G>T**	5′ss (10.1 → 3.2)	1.1% ± 0.2%	▼(E57q23b) ^3^ [1.9% ± 0.3%]	Δ(E57) [89.4% ± 1.7%]	1105-nt [7.6% ± 1.2%]
**c.8418+5_8418+8del**	5′ss (10.1 → 0.8)	-		Δ(E57) [96.1% ± 1.9%]	1105-nt [3.9% ± 1.9%]
**c.8584+1G>A**	5′ss (3.4 → −4.8)	-	Δ(E58q50) [34.7% ± 1.2%] Δ(E58q19) [60.5% ± 1.2%] ▼(E58q117a) ^3^ [4.8% ± 0.3%]		
**c.8584+2T>C**	5′ss (3.4 → −4.4)	-	Δ(E58q19) [41.1% ± 0.3%] Δ(E58q50) [27.2% ± 0.3%] Δ(E58) [12.3% ± 0.5%] ▼(E58q11) [7.4% ± 0.3%] ▼(E58q117b) ^3^ [4.1% ± 0.1%] [Δ(E58_E59)] [2.5% ± 0.8%] [Δ(E57_E58)] [2.3% ± 0.2%] ▼(E58q48) [1.5% ± 0.2%]		1089-nt [1.6% ± 0.1%]
**c.8584+4A>G**	5′ss (3.4 → −1.2)	63.4%	Δ(E58q50) [9.7% ± 0.3%] Δ(E58q19) [9.4% ± 0.1%] Δ(E58) [3.8% ± 0.3%] [Δ(E58_E59)] [1.8%] [Δ(E57_E58)] [1.3% ± 0.3%] ▼(E58q117c) ^3^ [1.2% ± 0.1%]		1187-nt [9.4% ± 0.3%]
**c.8671+1G>A**	5′ss (9.7 → 1.5)	-		Δ(E59) [100%]	
**c.8671+2T>A**	5′ss (9.7 → 1.5)	-		Δ(E59) [100%]	
mgATM_55–63_WT (58F+R)		100%			
**c.8672-3T>G**	3′ss (9.7 → 2.8)	57.1% ± 0.1%	Δ(E60p17) [42.9% ± 0.1%]		
**c.8786+1G>A**	5′SS (11 → 9.8)	-	Δ(E60) [88.8% ± 0.1%] ▼(E60q14) [11.2% ± 0.1%]		
**c.8850G>T**	5′ss (8.3 → 0.2)	-	Δ(E61) [100%]		
**c.8850+4A>C**	5′ss (8.3 → 6.6)	100%			
**c.8851-3T>G**	3′ss (11.3 → 1.9)	90.6% ± 0.2%		Δ(E62p63) [6.1%] Δ(E62p66) [1.4%]	595-nt [1.9% ± 0.2%]
**c.8851-1G>T**	3′ss (11.3 → 2.7)	-	Δ(E62p49) [75.1%]	Δ(E62p63) [17.2%] Δ(E62p66) [3.2%]	637-nt [4.5%]
**c.8851-1G>C**	3′ss (11.3 → 3.3)	-	Δ(E62p49) [69.4% ± 0.1%]	Δ(E62p63) [22.7% ± 0.1%] Δ(E62p66) [4.4% ± 0.2%]	637-nt [3.5%]
**c.8987+5G>C**	5′ss (9.6 → 6.5)	93.4% ± 0.8%	▼(I62) [2.5% ± 0.4%] ▼(E62q20) [1.6% ± 0.1%]		595-nt [2.5% ± 0.4%]
**c.8988-7_8988-5del**	3′ss (10.5 → 5.3)	100%			

^1^ Transcripts annotations: mgFL (canonical transcript generated by mg wt); Δ (skipping of the exonic sequences); ▼ (inclusion intronic sequences); E (exon), p (acceptor shift), q (donor shift). When necessary, the exact number of nt inserted or deleted is indicated. ^2^ PTC: premature termination codon; ^3^ The annotations a/b/c reflect the same splicing event with different sequences.

**Table 2:** ClinGen and minigene-based ACMG/AMP classification of 47 *ATM* variants.

*ATM* Variant		ClinVar		Proposed Minigene-Based ACMG/AMP Classification			
c.HGVS	p.HGVS	Class	Review Status ^1^	Class	Points	Minigene Readout Contribution	Others ACMG Evidences
**c.2839-2A>T**		P	*	VUS	(+2)	(+1)	(+1)
**c.2921C>T**	p.(Ser974Phe)	VUS	**	VUS	(+0)	(−1)	(+1)
**c.2922-1G>A**		P/LP	**	P	(+12)	(+8)	(+4)
**c.3077G>A**	p.(Trp1026*)	P/LP	**	P	(+11)	(+8)	(+3)
**c.3077+3A>C**		(-)	(-)	LB	(−5)	(−4)	(−1)
**c.3078-10T>G**		VUS	**	VUS	(+1)	(0)	(+1)
**c.3078-1G>A**		P	expert panel	P	(+14)	(+8)	(+6)
**c.3078G>T**	p.(Trp1026Cys)	P/LP	**	P	(+15)	(+8)	(+7)
**c.3153G>T**	p.(Glu1051Asp)	(-)	(-)	P	(+10)	(+8)	(+2)
**c.3153+4A>G**		VUS	**	LP	(+9)	(+8)	(+1)
**c.3154-7C>A**		(-)	(-)	LB	(−3)	(−4)	(+1)
**c.3154-6C>T**		(-)	(-)	LB	(−3)	(−4)	(+1)
**c.3284G>A**	p.(Arg1095Lys)	P	expert panel	P	(+12)	(+8)	(+4)
**c.3284G>C**	p.(Arg1095Thr)	P	expert panel	P	(+14)	(+8)	(+6)
**c.3284+1G>A**		LP	**	P	(+12)	(+8)	(+4)
**c.3284+4A>G**		VUS	**	P	(+12)	(+8)	(+4)
**c.6095G>A**	p.(Arg2032Lys)	P/LP	**	P	(+22)	(+8)	(+14)
**c.6095+4A>G**		P/LP	**	P	(+11)	(+8)	(+3)
**c.6095+6T>C**		LB	**	VUS	(+0)	(+0)	(+0)
**c.6096-2A>G**		P/LP	**	LP	(+9)	(+8)	(+1)
**c.6198+1G>A**		P/LP	**	P	(+14)	(+8)	(+6)
**c.6348-10T>A**		(-)	(-)	LB	(−3)	(−4)	(+1)
**c.6348-6_6348-5del**		(-)	(-)	LB	(−3)	(−4)	(+1)
**c.6348-2A>T**		(-)	(-)	P	(+11)	(+8)	(+3)
**c.6451A>G**	p.(Arg2151Gly)	(-)	(-)	P	(+10)	(+8)	(+2)
**c.8011-2A>C**		P	*	P	(+11)	(+8)	(+2)
**c.8011-2A>G**		P/LP	**	P	(+12)	(+8)	(+2)
**c.8152G>T**	p.(Gly2718Cys)	(-)	(-)	VUS	(0)	(+1)	(−1)
**c.8269-7A>G**		VUS	*	VUS	(+2)	(+1)	(+1)
**c.8269-2A>T**		P/LP	**	P	(+11)	(+8)	(+3)
**c.8418+5G>A**		P/LP	**	LP	(+9)	(+8)	(+1)
**c.8418+5G>T**		(-)	(-)	LP	(+9)	(+8)	(+1)
**c.8418+5_8418+8del**		P	**	P	(+11.5)	(+8)	(+3.5)
**c.8584+1G>A**		P/LP	**	P	(+12)	(+8)	(+4)
**c.8584+2T>C**		P/LP	**	P	(+13)	(+8)	(+5)
**c.8584+4A>G**		VUS	**	VUS	(+1)	(+0)	(+1)
**c.8671+1G>A**		LP	**	P	(+11)	(+8)	(+3)
**c.8671+2T>A**		(-)	(-)	P	(+11)	(+8)	(+3)
**c.8672-3T>G**		(-)	(-)	VUS	(+3)	(+0)	(+3)
**c.8786+1G>A**		P	expert panel	P	(+14)	(+8)	(+6)
**c.8850G>T**	p.(Glu2950Asp)	(-)	(-)	P	(+12)	(+8)	(+4)
**c.8850+4A>C**		VUS/LB	*	LB	(−6)	(−4)	(−2)
**c.8851-3T>G**		VUS/LB	*	LB	(−6)	(−4)	(−2)
**c.8851-1G>C**		LP/VUS	*	P	(+11)	(+8)	(+3)
**c.8851-1G>T**		P/LP	**	P	(+11)	(+8)	(+3)
**c.8987+5G>C**		VUS/LB	*	LB	(−5)	(−4)	(−1)
**c.8988-6_8988-4del**		VUS	*	LB	(−3)	(−4)	(+1)

The table compares the current ClinVar classification with the proposed point system minigene-based classification. Orange cells, pathogenic; pink cells, likely pathogenic; grey cells, VUS; Green cells, likely benign. ^1^ Review status on ClinVar: *, criteria provided, conflicting classifications or criteria provided, single submitter; **, criteria provided, multiple submitters, no conflicts. The contribution of minigene readouts and other ACMG/AMP evidences to the final classification is shown.

## Data Availability

All sequencing and fragment analysis data are available at Digital. CSIC: http://hdl.handle.net/10261/403423, accessed on 8 January 2026; http://doi.org/10.20350/DIGITALCSIC/17662, accessed on 8 January 2026.

## Supplementary Materials

The following supporting information can be downloaded at: https://www.mdpi.com/article/10.3390/ijms27020765/s1.

## Abbreviations

The following abbreviations are used in this manuscript:

ACMG/AMP	American College of Medical Genetics and Genomics/Association for Molecular Pathology
ATM	Ataxia–telangiectasia-mutated
BC	Breast Cancer
bp	Base pairs
BRIDGES	Breast Cancer After Diagnostic Gene Sequencing
DDR	DNA damage response
ER	estrogen receptor
HBOP VCEP	Hereditary Breast, Ovarian and Pancreatic Cancer Variant Curation Expert Panel
LB	Likely Benign
LP	Likely pathogenic
LoF	Loss-of-function
MAVE	Multiplexed assays of variant effects
MCF-7	Michigan Cancer Foundation-7
MDA-MB-231	M D Anderson-Metastatic Breast-231
MES	MaxEntScan
mgFL	minigene full-length
NMD	nonsense-mediated decay
P	Pathogenic
PTC	premature termination codon
PTV	protein-truncating variants
REVEL	Rare exome variant ensemble learner
RFU	Relative Fluorescence Units
RT-PCR	Reverse transcription polymerase chain reaction
SRE	Splicing Regulatory Elements
ss	Splice-site
VUS	Variants of Uncertain Significance
wt	Wild type
