# Supplementary material for: Experimental Mis-Splicing Assessment and ACMG/AMP-Guided Classification of 47 ATM Splice-Site Variants
Source: Int J Mol Sci. 2026 Jan 12;27(2):765. doi: 10.3390/ijms27020765 (PMC12840730; doi:10.3390/ijms27020765)
Supplement: Supplementary file 1 [file ijms-27-00765-s001.zip › Supplementary_Figure_S1_Splicing_Assays_Variants_ATM.pptx]

## Slide 1
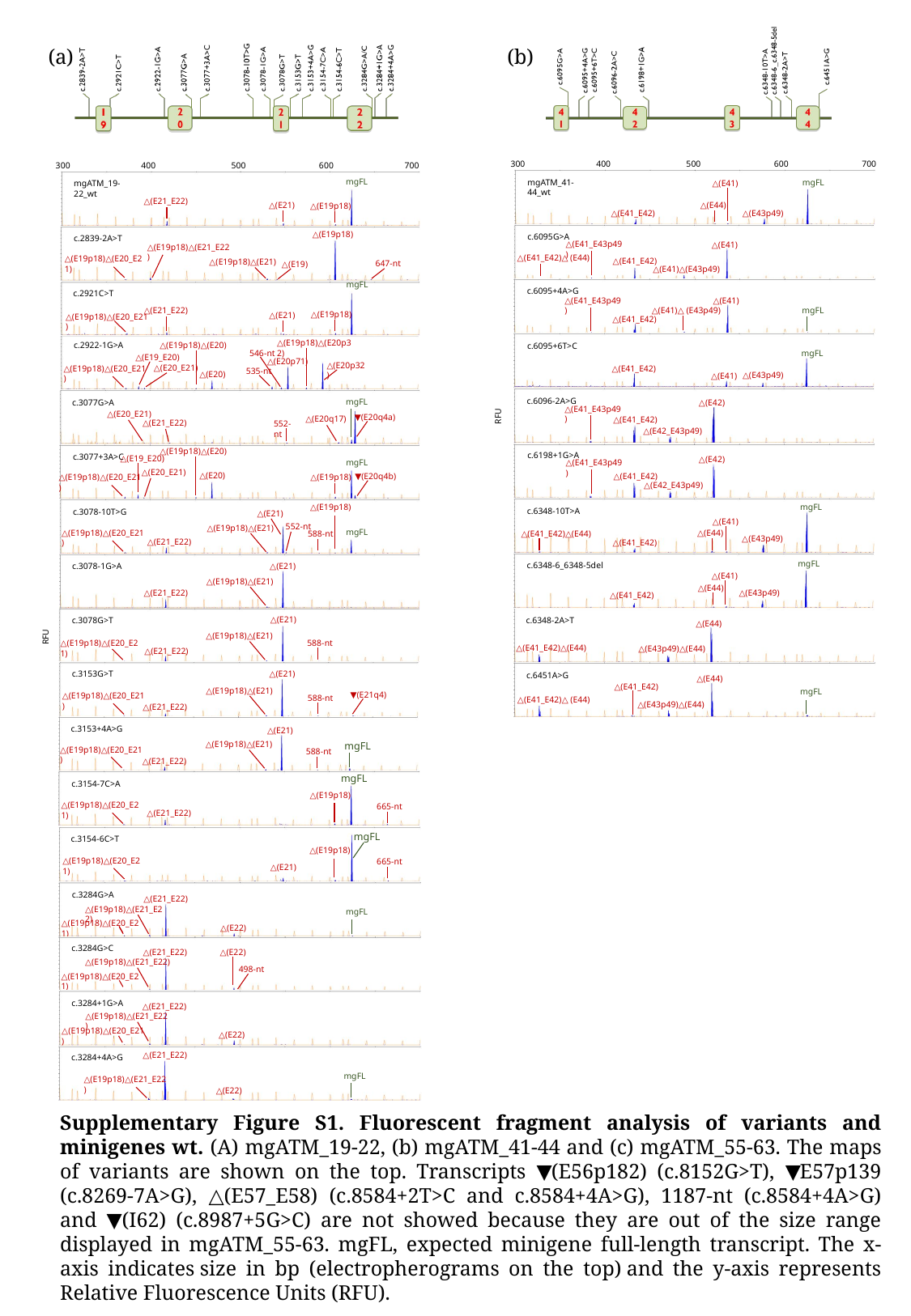

(a)
(b)
300 400 500 600 700
RFU
mgFL
mgATM_41-44_wt
△(E41)
△(E44)
△(E43p49)
△(E41_E42)
c.6095G>A
△(E41_E43p49)
△(E41)
△(E41_E42)△ (E44)
△(E41_E42)
△(E41)△(E43p49)
c.6095+4A>G
△(E41_E43p49)
△(E41)
mgFL
△(E41)△ (E43p49)
△(E41_E42)
c.6095+6T>C
mgFL
△(E41_E42)
△(E43p49)
△(E41)
c.6096-2A>G
△(E42)
△(E41_E43p49)
△(E41_E42)
△(E42_E43p49)
c.6198+1G>A
△(E42)
△(E41_E43p49)
△(E41_E42)
△(E42_E43p49)
mgFL
c.6348-10T>A
△(E41)
△(E44)
△(E41_E42)△(E44)
△(E43p49)
△(E41_E42)
mgFL
c.6348-6_6348-5del
△(E41)
△(E44)
△(E43p49)
△(E41_E42)
c.6348-2A>T
△(E44)
△(E41_E42)△(E44)
△(E43p49)△(E44)
c.6451A>G
△(E44)
△(E41_E42)
mgFL
△(E41_E42)△ (E44)
△(E43p49)△(E44)
300 400 500 600 700
mgFL
RFU
mgATM_19-22_wt
△(E21_E22)
△(E21)
△(E19p18)
△(E19p18)
c.2839-2A>T
△(E19p18)△(E21_E22)
△(E19p18)△(E20_E21)
△(E19p18)△(E21)
647-nt
△(E19)
mgFL
c.2921C>T
△(E21_E22)
△(E19p18)
△(E21)
△(E19p18)△(E20_E21)
△(E19p18)△(E20p32)
c.2922-1G>A
△(E19p18)△(E20)
546-nt
△(E19_E20)
△(E20p71)
△(E20p32)
△(E20_E21)
△(E19p18)△(E20_E21)
535-nt
△(E20)
mgFL
c.3077G>A
△(E20_E21)
▼(E20q4a)
△(E20q17)
△(E21_E22)
552-nt
△(E19p18)△(E20)
c.3077+3A>C
△(E19_E20)
mgFL
△(E20_E21)
△(E20)
▼(E20q4b)
△(E19p18)△(E20_E21)
△(E19p18)
△(E19p18)
c.3078-10T>G
△(E21)
552-nt
△(E19p18)△(E21)
mgFL
△(E19p18)△(E20_E21)
588-nt
△(E21_E22)
c.3078-1G>A
△(E21)
△(E19p18)△(E21)
△(E21_E22)
△(E21)
c.3078G>T
△(E19p18)△(E21)
△(E19p18)△(E20_E21)
588-nt
△(E21_E22)
c.3153G>T
△(E21)
△(E19p18)△(E21)
▼(E21q4)
△(E19p18)△(E20_E21)
588-nt
△(E21_E22)
c.3153+4A>G
△(E21)
△(E19p18)△(E21)
mgFL
△(E19p18)△(E20_E21)
588-nt
△(E21_E22)
mgFL
c.3154-7C>A
△(E19p18)
△(E19p18)△(E20_E21)
665-nt
△(E21_E22)
mgFL
c.3154-6C>T
△(E19p18)
△(E19p18)△(E20_E21)
665-nt
△(E21)
c.3284G>A
△(E21_E22)
△(E19p18)△(E21_E22)
mgFL
△(E19p18)△(E20_E21)
△(E22)
c.3284G>C
△(E21_E22)
△(E22)
△(E19p18)△(E21_E22)
498-nt
△(E19p18)△(E20_E21)
c.3284+1G>A
△(E21_E22)
△(E19p18)△(E21_E22)
△(E19p18)△(E20_E21)
△(E22)
△(E21_E22)
c.3284+4A>G
mgFL
△(E19p18)△(E21_E22)
△(E22)
Supplementary Figure S1. Fluorescent fragment analysis of variants and minigenes wt. (A) mgATM_19-22, (b) mgATM_41-44 and (c) mgATM_55-63. The maps of variants are shown on the top. Transcripts ▼(E56p182) (c.8152G>T), ▼E57p139 (c.8269-7A>G), △(E57_E58) (c.8584+2T>C and c.8584+4A>G), 1187-nt (c.8584+4A>G) and ▼(I62) (c.8987+5G>C) are not showed because they are out of the size range displayed in mgATM_55-63. mgFL, expected minigene full-length transcript. The x-axis indicates size in bp (electropherograms on the top) and the y-axis represents Relative Fluorescence Units (RFU).

## Slide 2
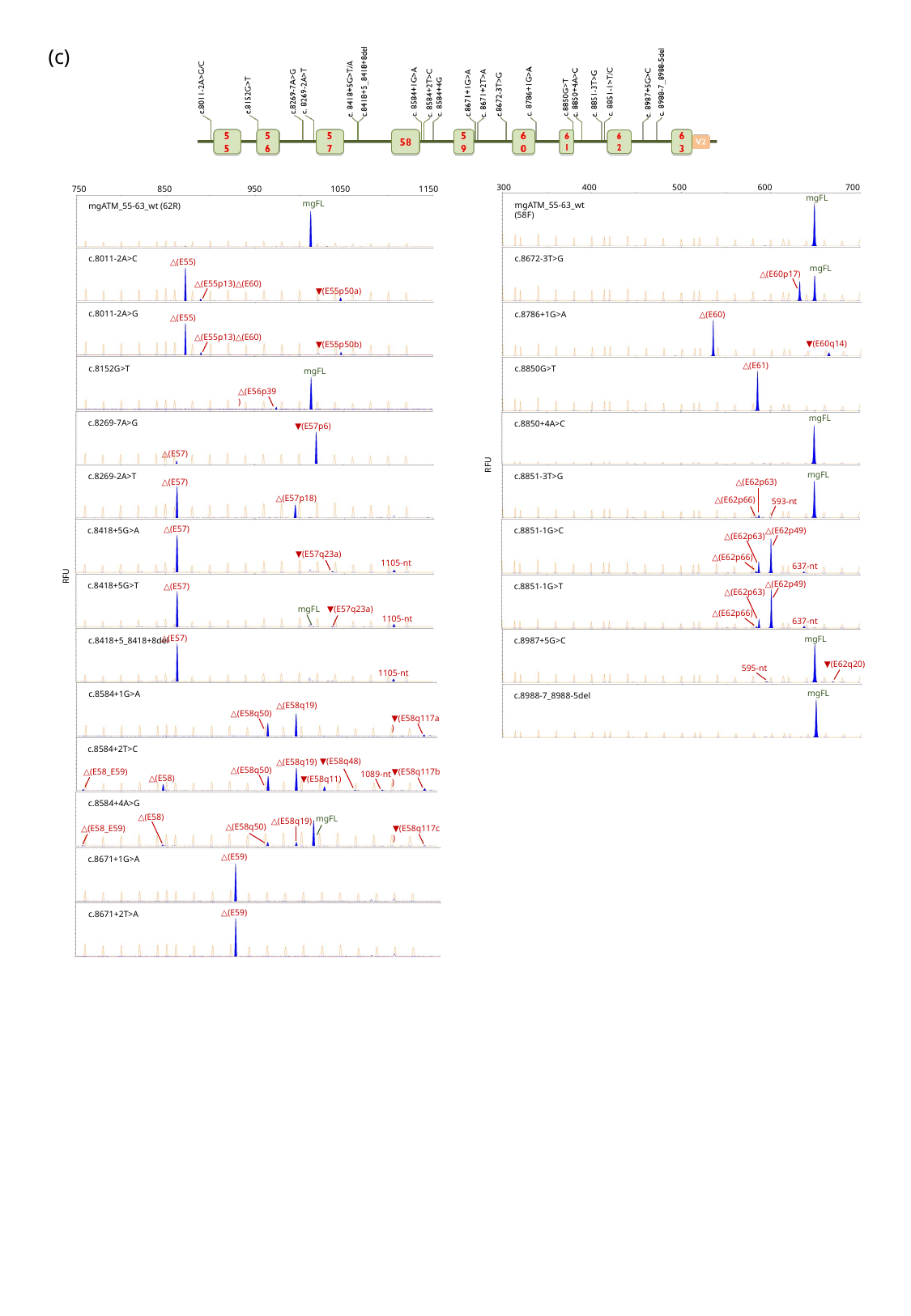

(c)
300 400 500 600 700
mgFL
RFU
mgATM_55-63_wt (58F)
c.8672-3T>G
mgFL
△(E60p17)
△(E60)
c.8786+1G>A
▼(E60q14)
△(E61)
c.8850G>T
mgFL
c.8850+4A>C
mgFL
c.8851-3T>G
△(E62p63)
△(E62p66)
593-nt
c.8851-1G>C
△(E62p49)
△(E62p63)
△(E62p66)
637-nt
△(E62p49)
c.8851-1G>T
△(E62p63)
△(E62p66)
637-nt
mgFL
c.8987+5G>C
▼(E62q20)
595-nt
mgFL
c.8988-7_8988-5del
750 850 950 1050 1150
mgFL
mgATM_55-63_wt (62R)
RFU
c.8011-2A>C
△(E55)
△(E55p13)△(E60)
▼(E55p50a)
c.8011-2A>G
△(E55)
△(E55p13)△(E60)
▼(E55p50b)
c.8152G>T
mgFL
△(E56p39)
c.8269-7A>G
▼(E57p6)
△(E57)
c.8269-2A>T
△(E57)
△(E57p18)
△(E57)
c.8418+5G>A
▼(E57q23a)
1105-nt
c.8418+5G>T
△(E57)
mgFL
▼(E57q23a)
1105-nt
△(E57)
c.8418+5_8418+8del
1105-nt
c.8584+1G>A
△(E58q19)
△(E58q50)
▼(E58q117a)
c.8584+2T>C
▼(E58q48)
△(E58q19)
△(E58q50)
△(E58_E59)
▼(E58q117b)
1089-nt
△(E58)
▼(E58q11)
c.8584+4A>G
△(E58)
mgFL
△(E58q19)
△(E58q50)
▼(E58q117c)
△(E58_E59)
△(E59)
c.8671+1G>A
△(E59)
c.8671+2T>A
