# Supplementary material for: Experimental Mis-Splicing Assessment and ACMG/AMP-Guided Classification of 47 ATM Splice-Site Variants
Source: Int J Mol Sci. 2026 Jan 12;27(2):765. doi: 10.3390/ijms27020765 (PMC12840730; doi:10.3390/ijms27020765)
Supplement: Supplementary file 1 [file ijms-27-00765-s001.zip › Supplementary_Figure_S2_Reproducibility of the minigenes analysis assays in MCF-7 and MDA-MD-231 cells.pptx]

## Slide 1
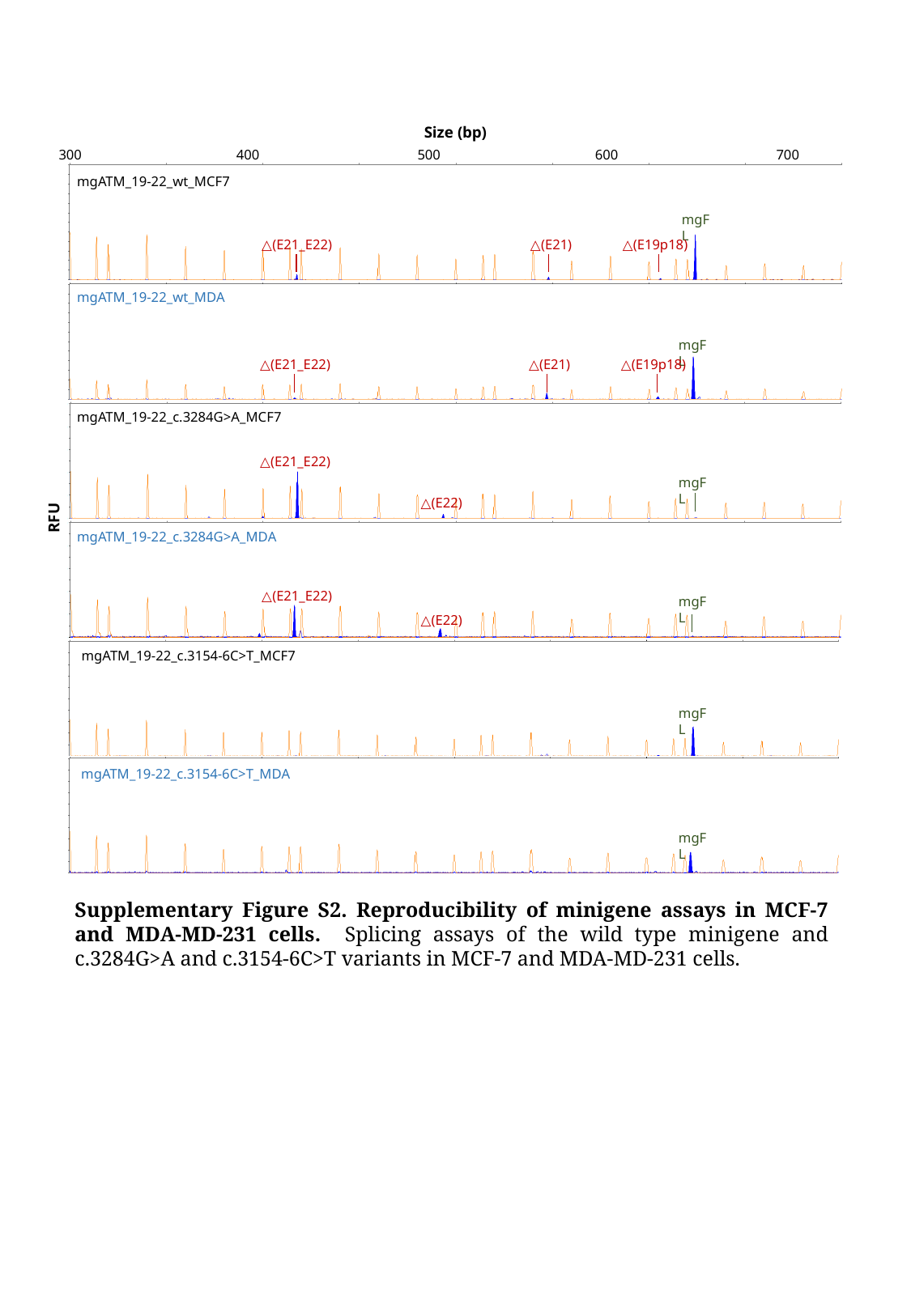

Size (bp)
300 400 500 600 700
RFU
mgATM_19-22_wt_MCF7
mgFL
△(E21_E22)
△(E21)
△(E19p18)
mgATM_19-22_wt_MDA
mgFL
△(E21_E22)
△(E21)
△(E19p18)
mgATM_19-22_c.3284G>A_MCF7
△(E21_E22)
mgFL
△(E22)
mgATM_19-22_c.3284G>A_MDA
△(E21_E22)
mgFL
△(E22)
mgATM_19-22_c.3154-6C>T_MCF7
mgFL
mgATM_19-22_c.3154-6C>T_MDA
mgFL
Supplementary Figure S2. Reproducibility of minigene assays in MCF-7 and MDA-MD-231 cells. Splicing assays of the wild type minigene and c.3284G>A and c.3154-6C>T variants in MCF-7 and MDA-MD-231 cells.
