# Supplementary material for: Experimental Mis-Splicing Assessment and ACMG/AMP-Guided Classification of 47 ATM Splice-Site Variants
Source: Int J Mol Sci. 2026 Jan 12;27(2):765. doi: 10.3390/ijms27020765 (PMC12840730; doi:10.3390/ijms27020765)
Supplement: Supplementary file 1 [file ijms-27-00765-s001.zip › Supplementary_Figure_S3_Insert Sequence Minigenes ATM.pptx]

## Slide 1
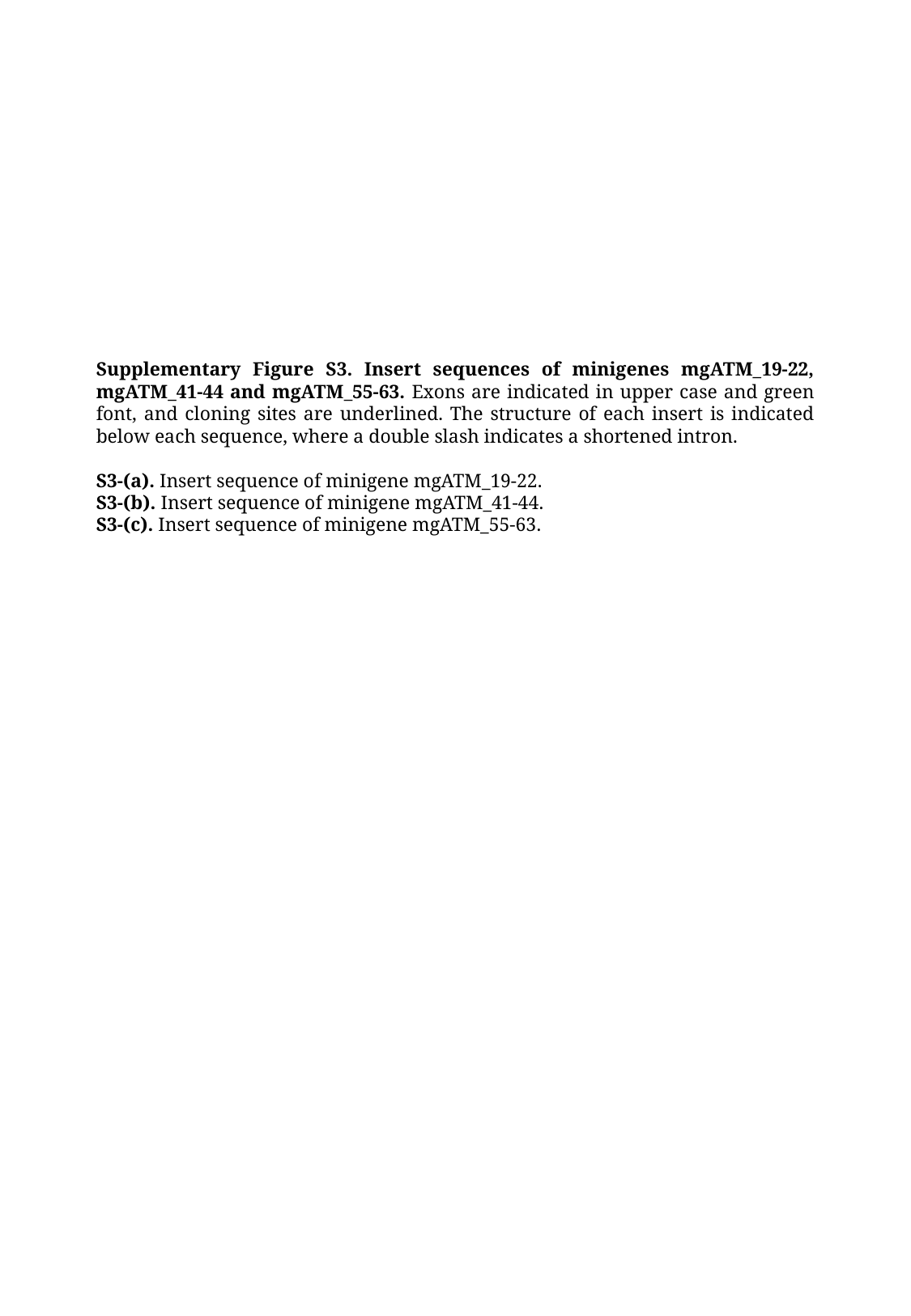

Supplementary Figure S3. Insert sequences of minigenes mgATM_19-22, mgATM_41-44 and mgATM_55-63. Exons are indicated in upper case and green font, and cloning sites are underlined. The structure of each insert is indicated below each sequence, where a double slash indicates a shortened intron.
S3-(a). Insert sequence of minigene mgATM_19-22.
S3-(b). Insert sequence of minigene mgATM_41-44.
S3-(c). Insert sequence of minigene mgATM_55-63.

## Slide 2
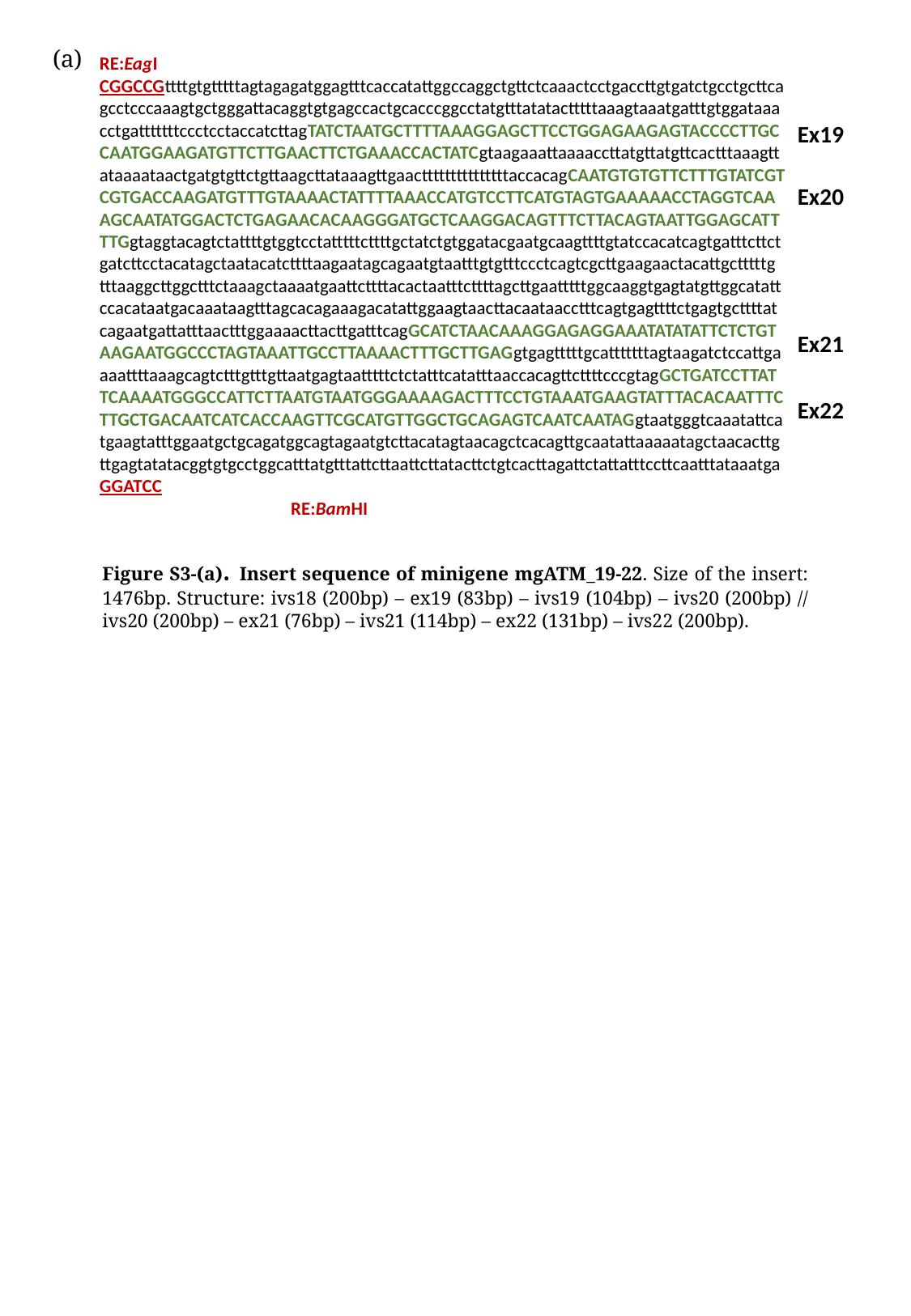

(a)
RE:EagI
CGGCCGttttgtgtttttagtagagatggagtttcaccatattggccaggctgttctcaaactcctgaccttgtgatctgcctgcttcagcctcccaaagtgctgggattacaggtgtgagccactgcacccggcctatgtttatatactttttaaagtaaatgatttgtggataaacctgatttttttccctcctaccatcttagTATCTAATGCTTTTAAAGGAGCTTCCTGGAGAAGAGTACCCCTTGCCAATGGAAGATGTTCTTGAACTTCTGAAACCACTATCgtaagaaattaaaaccttatgttatgttcactttaaagttataaaataactgatgtgttctgttaagcttataaagttgaactttttttttttttttaccacagCAATGTGTGTTCTTTGTATCGTCGTGACCAAGATGTTTGTAAAACTATTTTAAACCATGTCCTTCATGTAGTGAAAAACCTAGGTCAAAGCAATATGGACTCTGAGAACACAAGGGATGCTCAAGGACAGTTTCTTACAGTAATTGGAGCATTTTGgtaggtacagtctattttgtggtcctatttttcttttgctatctgtggatacgaatgcaagttttgtatccacatcagtgatttcttctgatcttcctacatagctaatacatcttttaagaatagcagaatgtaatttgtgtttccctcagtcgcttgaagaactacattgctttttgtttaaggcttggctttctaaagctaaaatgaattcttttacactaatttcttttagcttgaatttttggcaaggtgagtatgttggcatattccacataatgacaaataagtttagcacagaaagacatattggaagtaacttacaataacctttcagtgagttttctgagtgcttttatcagaatgattatttaactttggaaaacttacttgatttcagGCATCTAACAAAGGAGAGGAAATATATATTCTCTGTAAGAATGGCCCTAGTAAATTGCCTTAAAACTTTGCTTGAGgtgagtttttgcatttttttagtaagatctccattgaaaattttaaagcagtctttgtttgttaatgagtaatttttctctatttcatatttaaccacagttcttttcccgtagGCTGATCCTTATTCAAAATGGGCCATTCTTAATGTAATGGGAAAAGACTTTCCTGTAAATGAAGTATTTACACAATTTCTTGCTGACAATCATCACCAAGTTCGCATGTTGGCTGCAGAGTCAATCAATAGgtaatgggtcaaatattcatgaagtatttggaatgctgcagatggcagtagaatgtcttacatagtaacagctcacagttgcaatattaaaaatagctaacacttgttgagtatatacggtgtgcctggcatttatgtttattcttaattcttatacttctgtcacttagattctattatttccttcaatttataaatgaGGATCC
 RE:BamHI
Ex19
Ex20
Ex21
Ex22
Figure S3-(a). Insert sequence of minigene mgATM_19-22. Size of the insert: 1476bp. Structure: ivs18 (200bp) – ex19 (83bp) – ivs19 (104bp) – ivs20 (200bp) // ivs20 (200bp) – ex21 (76bp) – ivs21 (114bp) – ex22 (131bp) – ivs22 (200bp).

## Slide 3
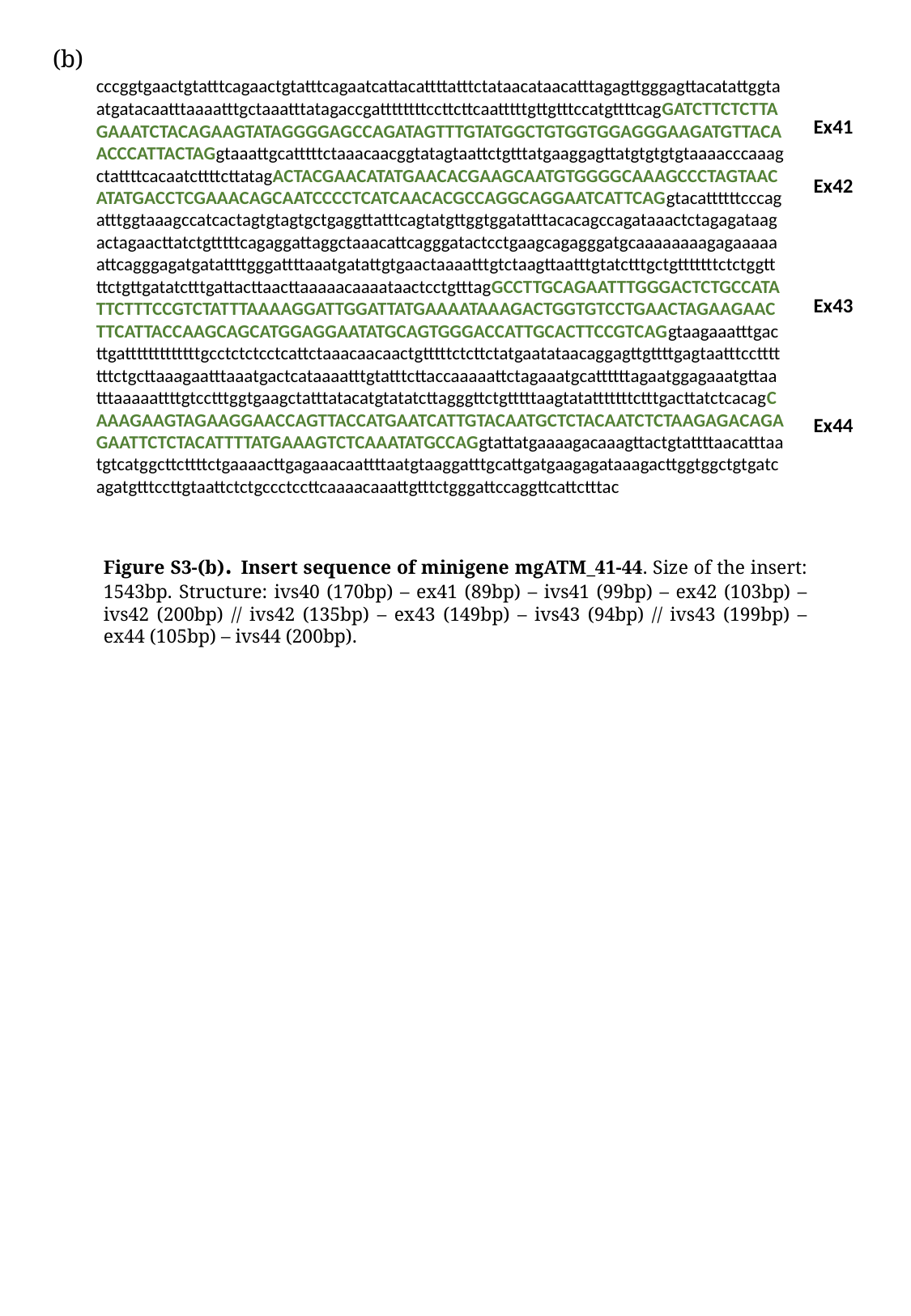

(b)
cccggtgaactgtatttcagaactgtatttcagaatcattacattttatttctataacataacatttagagttgggagttacatattggtaatgatacaatttaaaatttgctaaatttatagaccgattttttttccttcttcaatttttgttgtttccatgttttcagGATCTTCTCTTAGAAATCTACAGAAGTATAGGGGAGCCAGATAGTTTGTATGGCTGTGGTGGAGGGAAGATGTTACAACCCATTACTAGgtaaattgcatttttctaaacaacggtatagtaattctgtttatgaaggagttatgtgtgtgtaaaacccaaagctattttcacaatcttttcttatagACTACGAACATATGAACACGAAGCAATGTGGGGCAAAGCCCTAGTAACATATGACCTCGAAACAGCAATCCCCTCATCAACACGCCAGGCAGGAATCATTCAGgtacattttttcccagatttggtaaagccatcactagtgtagtgctgaggttatttcagtatgttggtggatatttacacagccagataaactctagagataagactagaacttatctgtttttcagaggattaggctaaacattcagggatactcctgaagcagagggatgcaaaaaaaagagaaaaaattcagggagatgatattttgggattttaaatgatattgtgaactaaaatttgtctaagttaatttgtatctttgctgtttttttctctggttttctgttgatatctttgattacttaacttaaaaacaaaataactcctgtttagGCCTTGCAGAATTTGGGACTCTGCCATATTCTTTCCGTCTATTTAAAAGGATTGGATTATGAAAATAAAGACTGGTGTCCTGAACTAGAAGAACTTCATTACCAAGCAGCATGGAGGAATATGCAGTGGGACCATTGCACTTCCGTCAGgtaagaaatttgacttgatttttttttttttgcctctctcctcattctaaacaacaactgtttttctcttctatgaatataacaggagttgttttgagtaatttcctttttttctgcttaaagaatttaaatgactcataaaatttgtatttcttaccaaaaattctagaaatgcattttttagaatggagaaatgttaatttaaaaattttgtcctttggtgaagctatttatacatgtatatcttagggttctgtttttaagtatatttttttctttgacttatctcacagCAAAGAAGTAGAAGGAACCAGTTACCATGAATCATTGTACAATGCTCTACAATCTCTAAGAGACAGAGAATTCTCTACATTTTATGAAAGTCTCAAATATGCCAGgtattatgaaaagacaaagttactgtattttaacatttaatgtcatggcttcttttctgaaaacttgagaaacaattttaatgtaaggatttgcattgatgaagagataaagacttggtggctgtgatcagatgtttccttgtaattctctgccctccttcaaaacaaattgtttctgggattccaggttcattctttac
Ex41
Ex42
Ex43
Ex44
Figure S3-(b). Insert sequence of minigene mgATM_41-44. Size of the insert: 1543bp. Structure: ivs40 (170bp) – ex41 (89bp) – ivs41 (99bp) – ex42 (103bp) – ivs42 (200bp) // ivs42 (135bp) – ex43 (149bp) – ivs43 (94bp) // ivs43 (199bp) – ex44 (105bp) – ivs44 (200bp).

## Slide 4
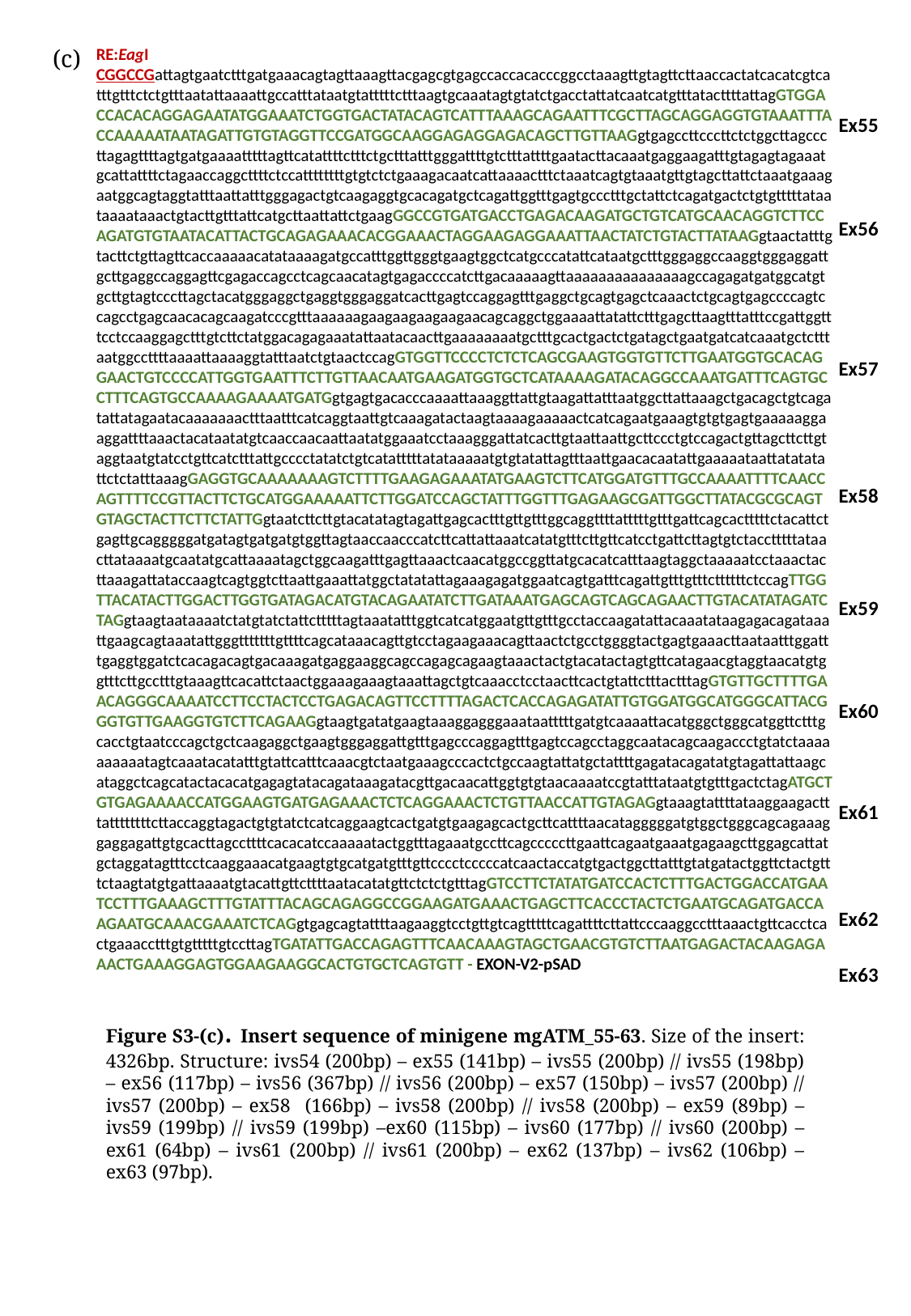

RE:EagI
CGGCCGattagtgaatctttgatgaaacagtagttaaagttacgagcgtgagccaccacacccggcctaaagttgtagttcttaaccactatcacatcgtcatttgtttctctgtttaatattaaaattgccatttataatgtatttttctttaagtgcaaatagtgtatctgacctattatcaatcatgtttatacttttattagGTGGACCACACAGGAGAATATGGAAATCTGGTGACTATACAGTCATTTAAAGCAGAATTTCGCTTAGCAGGAGGTGTAAATTTACCAAAAATAATAGATTGTGTAGGTTCCGATGGCAAGGAGAGGAGACAGCTTGTTAAGgtgagccttcccttctctggcttagcccttagagttttagtgatgaaaatttttagttcatattttctttctgctttatttgggattttgtctttattttgaatacttacaaatgaggaagatttgtagagtagaaatgcattattttctagaaccaggcttttctccattttttttgtgtctctgaaagacaatcattaaaactttctaaatcagtgtaaatgttgtagcttattctaaatgaaagaatggcagtaggtatttaattatttgggagactgtcaagaggtgcacagatgctcagattggtttgagtgccctttgctattctcagatgactctgtgtttttataataaaataaactgtacttgtttattcatgcttaattattctgaagGGCCGTGATGACCTGAGACAAGATGCTGTCATGCAACAGGTCTTCCAGATGTGTAATACATTACTGCAGAGAAACACGGAAACTAGGAAGAGGAAATTAACTATCTGTACTTATAAGgtaactatttgtacttctgttagttcaccaaaaacatataaaagatgccatttggttgggtgaagtggctcatgcccatattcataatgctttgggaggccaaggtgggaggattgcttgaggccaggagttcgagaccagcctcagcaacatagtgagaccccatcttgacaaaaagttaaaaaaaaaaaaaaagccagagatgatggcatgtgcttgtagtcccttagctacatgggaggctgaggtgggaggatcacttgagtccaggagtttgaggctgcagtgagctcaaactctgcagtgagccccagtccagcctgagcaacacagcaagatcccgtttaaaaaagaagaagaagaagaacagcaggctggaaaattatattctttgagcttaagtttatttccgattggtttcctccaaggagctttgtcttctatggacagagaaatattaatacaacttgaaaaaaaatgctttgcactgactctgatagctgaatgatcatcaaatgctctttaatggccttttaaaattaaaaggtatttaatctgtaactccagGTGGTTCCCCTCTCTCAGCGAAGTGGTGTTCTTGAATGGTGCACAGGAACTGTCCCCATTGGTGAATTTCTTGTTAACAATGAAGATGGTGCTCATAAAAGATACAGGCCAAATGATTTCAGTGCCTTTCAGTGCCAAAAGAAAATGATGgtgagtgacacccaaaattaaaggttattgtaagattatttaatggcttattaaagctgacagctgtcagatattatagaatacaaaaaaactttaatttcatcaggtaattgtcaaagatactaagtaaaagaaaaactcatcagaatgaaagtgtgtgagtgaaaaaggaaggattttaaactacataatatgtcaaccaacaattaatatggaaatcctaaagggattatcacttgtaattaattgcttccctgtccagactgttagcttcttgtaggtaatgtatcctgttcatctttattgcccctatatctgtcatatttttatataaaaatgtgtatattagtttaattgaacacaatattgaaaaataattatatatattctctatttaaagGAGGTGCAAAAAAAGTCTTTTGAAGAGAAATATGAAGTCTTCATGGATGTTTGCCAAAATTTTCAACCAGTTTTCCGTTACTTCTGCATGGAAAAATTCTTGGATCCAGCTATTTGGTTTGAGAAGCGATTGGCTTATACGCGCAGTGTAGCTACTTCTTCTATTGgtaatcttcttgtacatatagtagattgagcactttgttgtttggcaggttttatttttgtttgattcagcactttttctacattctgagttgcagggggatgatagtgatgatgtggttagtaaccaacccatcttcattattaaatcatatgtttcttgttcatcctgattcttagtgtctacctttttataacttataaaatgcaatatgcattaaaatagctggcaagatttgagttaaactcaacatggccggttatgcacatcatttaagtaggctaaaaatcctaaactacttaaagattataccaagtcagtggtcttaattgaaattatggctatatattagaaagagatggaatcagtgatttcagattgtttgtttcttttttctccagTTGGTTACATACTTGGACTTGGTGATAGACATGTACAGAATATCTTGATAAATGAGCAGTCAGCAGAACTTGTACATATAGATCTAGgtaagtaataaaatctatgtatctattctttttagtaaatatttggtcatcatggaatgttgtttgcctaccaagatattacaaatataagagacagataaattgaagcagtaaatattgggtttttttgttttcagcataaacagttgtcctagaagaaacagttaactctgcctggggtactgagtgaaacttaataatttggatttgaggtggatctcacagacagtgacaaagatgaggaaggcagccagagcagaagtaaactactgtacatactagtgttcatagaacgtaggtaacatgtggtttcttgcctttgtaaagttcacattctaactggaaagaaagtaaattagctgtcaaacctcctaacttcactgtattctttactttagGTGTTGCTTTTGAACAGGGCAAAATCCTTCCTACTCCTGAGACAGTTCCTTTTAGACTCACCAGAGATATTGTGGATGGCATGGGCATTACGGGTGTTGAAGGTGTCTTCAGAAGgtaagtgatatgaagtaaaggagggaaataatttttgatgtcaaaattacatgggctgggcatggttctttgcacctgtaatcccagctgctcaagaggctgaagtgggaggattgtttgagcccaggagtttgagtccagcctaggcaatacagcaagaccctgtatctaaaaaaaaaatagtcaaatacatatttgtattcatttcaaacgtctaatgaaagcccactctgccaagtattatgctattttgagatacagatatgtagattattaagcataggctcagcatactacacatgagagtatacagataaagatacgttgacaacattggtgtgtaacaaaatccgtatttataatgtgtttgactctagATGCTGTGAGAAAACCATGGAAGTGATGAGAAACTCTCAGGAAACTCTGTTAACCATTGTAGAGgtaaagtattttataaggaagactttattttttttcttaccaggtagactgtgtatctcatcaggaagtcactgatgtgaagagcactgcttcattttaacatagggggatgtggctgggcagcagaaaggaggagattgtgcacttagccttttcacacatccaaaaatactggtttagaaatgccttcagcccccttgaattcagaatgaaatgagaagcttggagcattatgctaggatagtttcctcaaggaaacatgaagtgtgcatgatgtttgttcccctcccccatcaactaccatgtgactggcttatttgtatgatactggttctactgtttctaagtatgtgattaaaatgtacattgttcttttaatacatatgttctctctgtttagGTCCTTCTATATGATCCACTCTTTGACTGGACCATGAATCCTTTGAAAGCTTTGTATTTACAGCAGAGGCCGGAAGATGAAACTGAGCTTCACCCTACTCTGAATGCAGATGACCAAGAATGCAAACGAAATCTCAGgtgagcagtattttaagaaggtcctgttgtcagtttttcagattttcttattcccaaggcctttaaactgttcacctcactgaaacctttgtgtttttgtccttagTGATATTGACCAGAGTTTCAACAAAGTAGCTGAACGTGTCTTAATGAGACTACAAGAGAAACTGAAAGGAGTGGAAGAAGGCACTGTGCTCAGTGTT - EXON-V2-pSAD
(c)
Ex55
Ex56
Ex57
Ex58
Ex59
Ex60
Ex61
Ex62
Ex63
Figure S3-(c). Insert sequence of minigene mgATM_55-63. Size of the insert: 4326bp. Structure: ivs54 (200bp) – ex55 (141bp) – ivs55 (200bp) // ivs55 (198bp) – ex56 (117bp) – ivs56 (367bp) // ivs56 (200bp) – ex57 (150bp) – ivs57 (200bp) // ivs57 (200bp) – ex58 (166bp) – ivs58 (200bp) // ivs58 (200bp) – ex59 (89bp) – ivs59 (199bp) // ivs59 (199bp) –ex60 (115bp) – ivs60 (177bp) // ivs60 (200bp) – ex61 (64bp) – ivs61 (200bp) // ivs61 (200bp) – ex62 (137bp) – ivs62 (106bp) – ex63 (97bp).
