# Supplementary material for: Experimental Mis-Splicing Assessment and ACMG/AMP-Guided Classification of 47 ATM Splice-Site Variants
Source: Int J Mol Sci. 2026 Jan 12;27(2):765. doi: 10.3390/ijms27020765 (PMC12840730; doi:10.3390/ijms27020765)
Supplement: Supplementary file 1 [file ijms-27-00765-s001.zip › Supplementary_Figure_S4_PCR_cycles.pptx]

## Slide 1
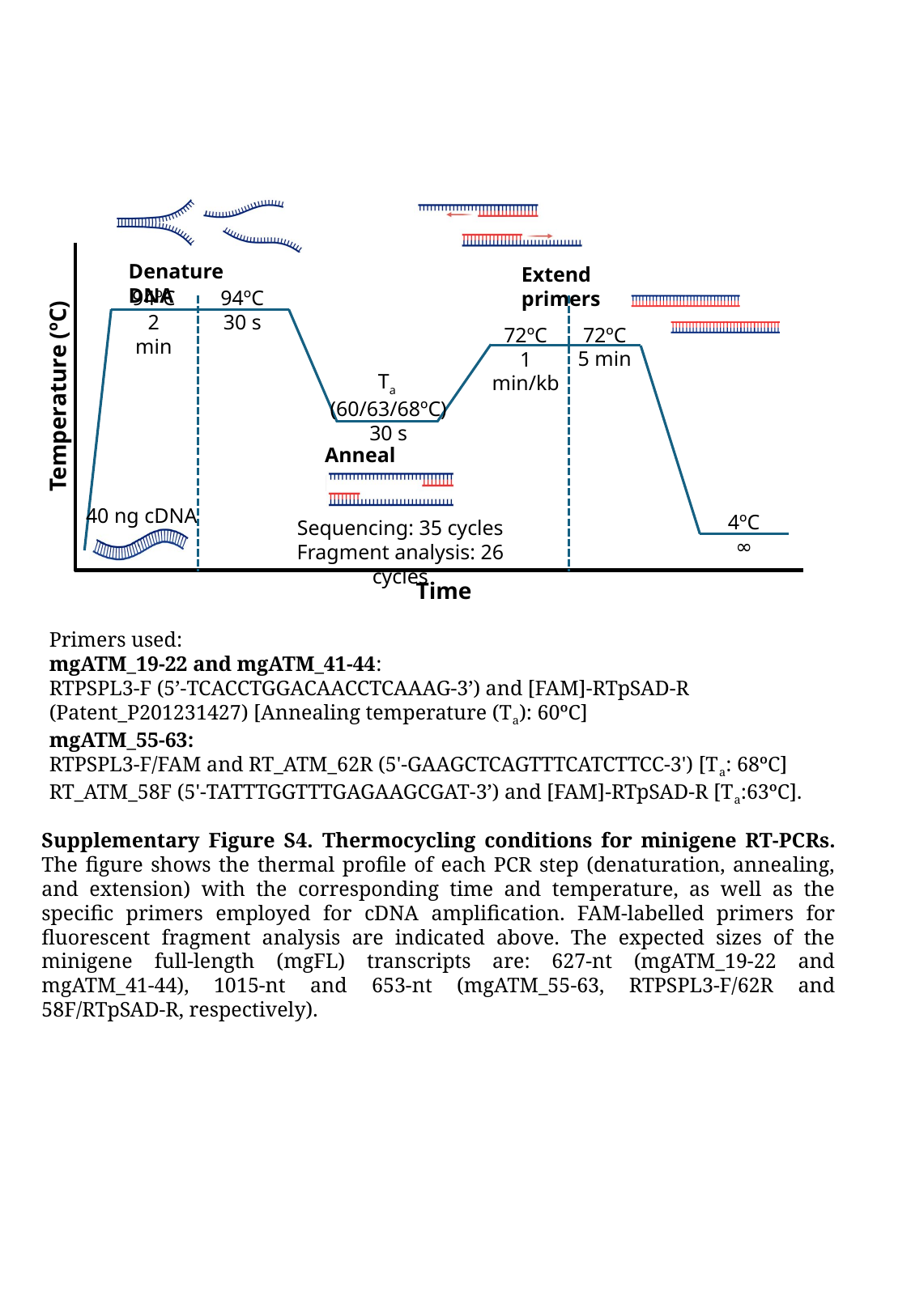

Denature DNA
Extend primers
94ºC
2 min
94ºC
30 s
72ºC
1 min/kb
72ºC
5 min
Ta
(60/63/68ºC)
30 s
Temperature (ºC)
Anneal primers
40 ng cDNA
4ºC
∞
Sequencing: 35 cycles
Fragment analysis: 26 cycles
Time
Primers used:
mgATM_19-22 and mgATM_41-44:
RTPSPL3-F (5’-TCACCTGGACAACCTCAAAG-3’) and [FAM]-RTpSAD-R (Patent_P201231427) [Annealing temperature (Ta): 60ºC]
mgATM_55-63:
RTPSPL3-F/FAM and RT_ATM_62R (5'-GAAGCTCAGTTTCATCTTCC-3') [Ta: 68ºC]
RT_ATM_58F (5'-TATTTGGTTTGAGAAGCGAT-3’) and [FAM]-RTpSAD-R [Ta:63ºC].
Supplementary Figure S4. Thermocycling conditions for minigene RT-PCRs. The figure shows the thermal profile of each PCR step (denaturation, annealing, and extension) with the corresponding time and temperature, as well as the specific primers employed for cDNA amplification. FAM-labelled primers for fluorescent fragment analysis are indicated above. The expected sizes of the minigene full-length (mgFL) transcripts are: 627-nt (mgATM_19-22 and mgATM_41-44), 1015-nt and 653-nt (mgATM_55-63, RTPSPL3-F/62R and 58F/RTpSAD-R, respectively).
