# Supplementary material for: Experimental Mis-Splicing Assessment and ACMG/AMP-Guided Classification of 47 ATM Splice-Site Variants
Source: Int J Mol Sci. 2026 Jan 12;27(2):765. doi: 10.3390/ijms27020765 (PMC12840730; doi:10.3390/ijms27020765)
Supplement: Supplementary file 1 [file ijms-27-00765-s001.zip › Supplementary_Table_S1_Transcript_Annotation_HGVS.pdf]

**Supplementary Table S1.** Enigma and HGVS annotations according to the MANE select transcript NM_000051.4 (ENST00000675843.1)

| *ATM* transcripts | HGVS-RNA | HGVS-protein | Diagrams of the splicing events |
|---|---|---|---|
| mgFL<br>mgATM_19-22 | | | 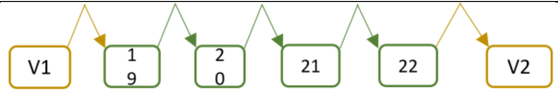  |
| mgFL c.2921C>T | r.2921c>u | p.(Ser974Phe) | |
| mgFL c.3077G>A | r.3077g>a<br>PTC-NMD | p.(Trp1026*) | |
| mgFL c.3284G>A | r.3284g>a | p.(Arg1095Lys) | |
| △(E19p18) | r.2839_2856del | p.(Tyr947_Lys952del) | 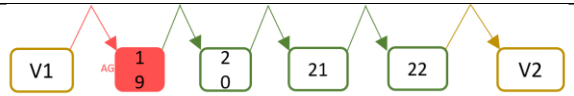  |
| △(E19) | r.2839_2921del<br>PTC-NMD | p.(Tyr947Glnfs*9) | 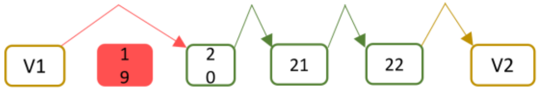  |
| △(E20p32) | r.2922_2953del<br>PTC-NMD | p.(Asn975Cysfs*3) | 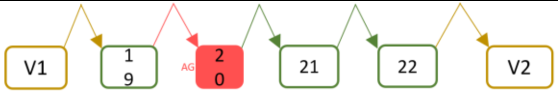  |
| △(E20p71) | r.2922_2992del<br>PTC-NMD | p.(Asn975Glufs*11) | 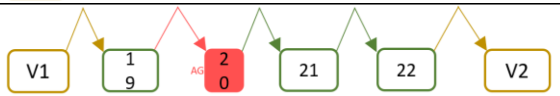  |
| △(E20) | r.2922_3077del | p.(Asn975_Trp1026del) | 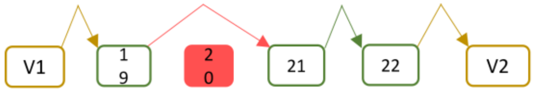  |
| △(E19_E20) | r.2839_3077del<br>PTC-NMD | p.(Tyr947Alafs*21) | 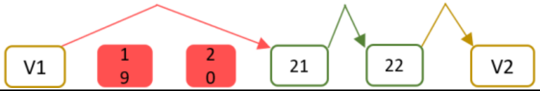  |
| △(E19p18)△(E20p32) | r.[2839_2856del;2922_2953del]<br>PTC-NMD | p.([Tyr947_Lys952del;<br>Asn975Cysfs*3]) | 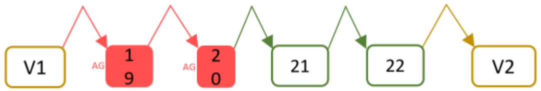  |
| △(E19p18)△(E20) | r.[2839_2856del;2922_3077del] | p.([Tyr947_Trp1026del;<br>Asn975_Trp1026del]) | 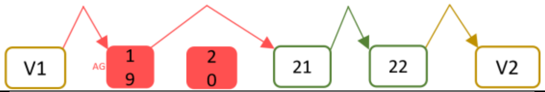  |
| △(E20q17) | r.3061_3077del<br>PTC-NMD | p.(Val1021Alafs*21) | 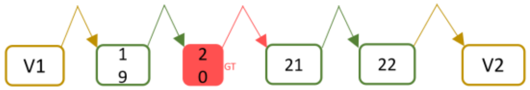  |
| ▼(E20q4a)[1] | r.3077_3078ins[a;3077+1_3077+4]<br>PTC-NMD | p.(Trp1026*) | 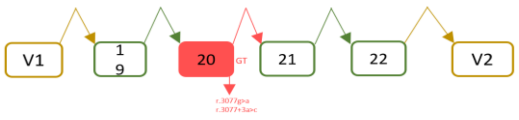  |
| ▼(E20q4b)[1] | r.3077_3078ins[3077+1_3077+2;c;3077+4]<br>PTC-NMD | p.(His1027Serfs*22) | |

| | | | |
|---|---|---|---|
| △(E21) | r.3078_3153del<br>PTC-NMD | p.(His1027Leufs*12) | 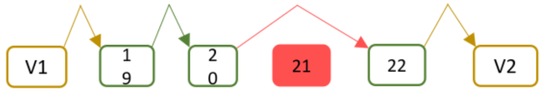 |
| ▼(E21q4) | r.3153_3154ins[u;3153+1_3153+4]<br>PTC-NMD | p.(Glu1051Aspfs*3) | 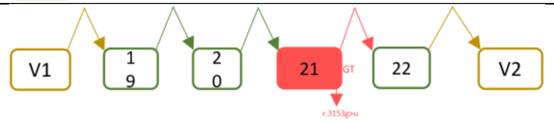 |
| △(E20_E21) | r.2922_3153del<br>PTC-NMD | p.(Asn975Leufs*12) | 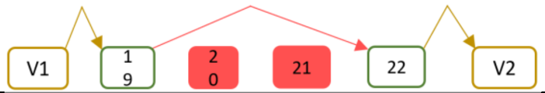 |
| △(E19p18)△(E21) | r.[2839_2856del; 3078_3153del]<br>PTC-NMD | p.([Tyr947_Lys952del;<br>His1027Leufs*12]) | 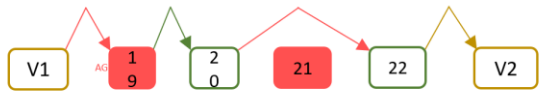 |
| △(E19p18)△(E20_E21) | r.[2839_2856del; 2922_3153del]<br>PTC-NMD | p.([Tyr947_Lys952del;<br>Asn975Leufs*12]) | 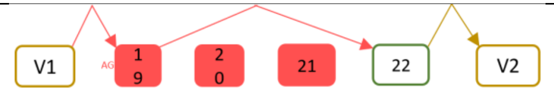 |
| △(E22) | r.3154_3284del<br>PTC-NMD | p.(Ala1052Ilefs*26) | 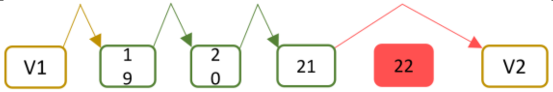 |
| △(E21_E22) | r.3078_3284del<br>PTC-NMD | p.(Trp1026*) | 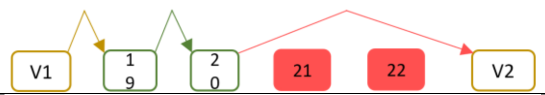 |
| △(E19p18)△(E21_E22) | r.[2839_2856del; 3078_3284del]<br>PTC-NMD | p.([Tyr947_Lys952del;<br>Trp1026*]) | 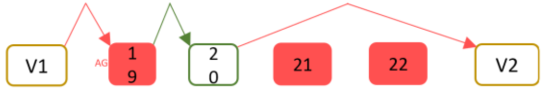 |
| mgFL<br>mgATM_41-44 | | | 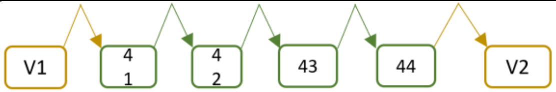 |
| mgFL c.6451A>G | r.6451a>g | p.(Arg2151Gly) | |
| △(E41) | r.6007_6095del<br>PTC-NMD | p.(Asp2003Thrfs*5) | 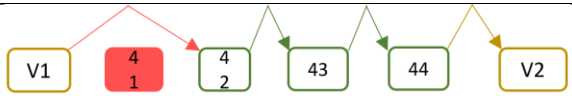 |
| △(E42) | r.6096_6198del<br>PTC-NMD | p.(Leu2033Profs*15) | 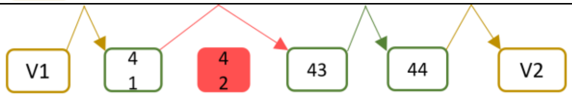 |
| △(E41_E42) | r.6007_6198del | p.(Asp2003_Gln2066del) | 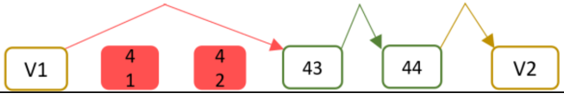 |

| | | | |
|---|---|---|---|
| △(E43p49) | r.6199_6247del<br>PTC-NMD | p.(Ala2067Aspfs*13) | 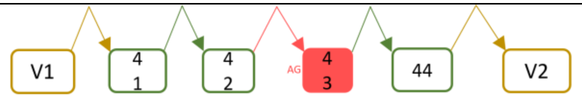  |
| △(E41)△(E43p49) | r.[6007_6095del; 6199_6247del]<br>PTC-NMD | p.(Asp2003Thrfs*5) | 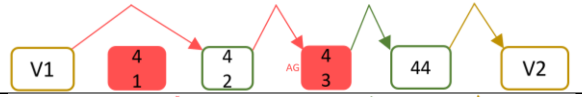  |
| △(E41_E43p49) | r.6007_6247del<br>PTC-NMD | p.(Leu2004Trpfs*12) | 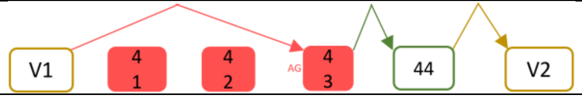  |
| △(E42_E43p49) | r.6096_6247del<br>PTC-NMD | p.(Leu2033Ilefs*4) | 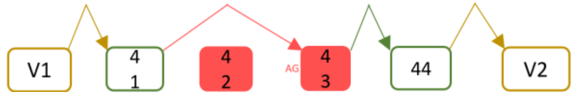  |
| △(E44) | r.6348_6452del | p.(Ser2116_Ala2150del) | 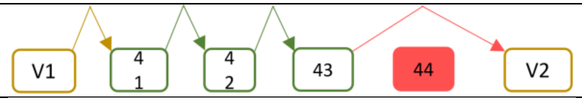  |
| △(E43p49)△(E44) | r.[6199_6247del; 6348_6452del]<br>PTC-NMD | p.(Ala2067Aspfs*13) | 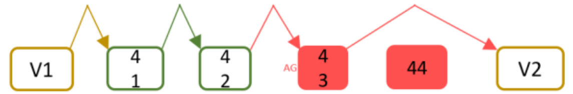  |
| △(E41_E42)△(E44) | r.[6007_6198del;6348_6452del] | p.([Asp2003_Gln2066del,<br>Ser2116_Ala2150del]) | 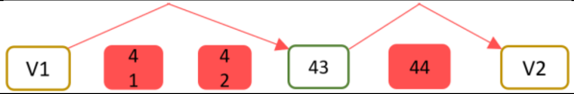  |
| mgFL<br>mgATM_55-63_WT | | | 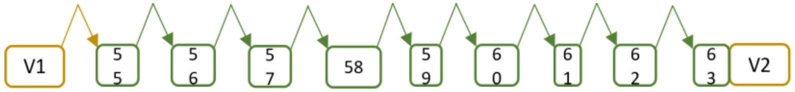  |
| ▼(E55p50a)[1] | r.8010_8011ins[8011-50_8011-3;c;8011-2_8011-1]<br>PTC-NMD | p.(Val2671Cysfs*3) | 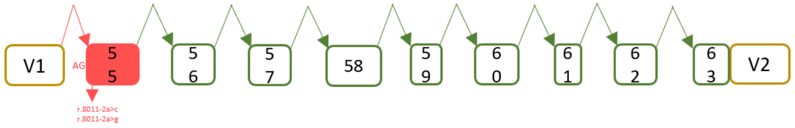  |
| ▼(E55p50b)[1] | r.8010_8011ins[8011-50_8011-3;g;8011-2_8011-1]<br>PTC-NMD | p.(Val2671Cysfs*3) | |
| △(E55p13)△(E60) | r.[8011_8023del;8672_8786del]<br>PTC-NMD | p.(Val2671Glufs*7) | 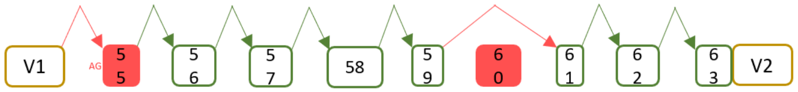  |
| △(E55) | r.8011_8151del | p.(Val2671_Lys2717del) | 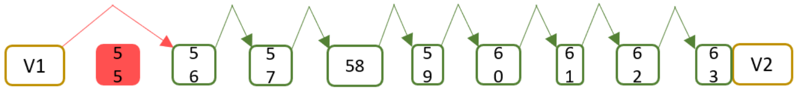  |
| mgFL c.8152G>T | r.8152g>u | p.(Gly2718Cys) | |

| | | | |
|---|---|---|---|
| △(E56p39) | r.8152_8190del | p.(Gly2718_Gln2730del) | 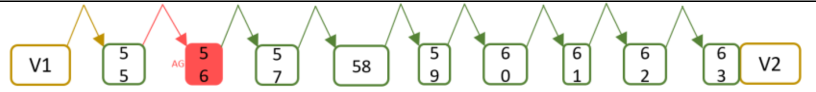 |
| ▼(E56p182) | r.8151_8152ins[8152-182_8152-1;u]<br>PTC-NMD | p.(Gly2718Cysfs*8) | 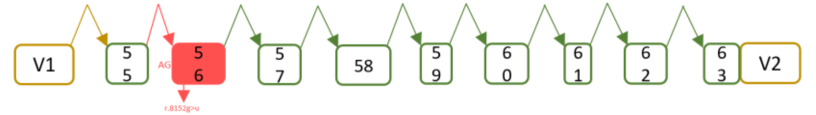 |
| ▼(E57p139) | r.8268_8269ins[8269-139_8269-8;g;8269-6_8269-1]<br>PTC-NMD | p.(Lys2756fs*11) | 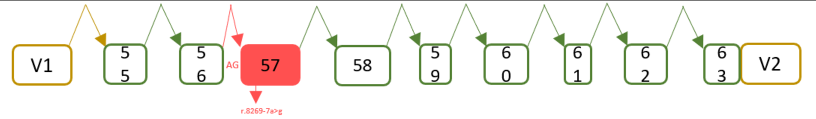 |
| ▼(E57p6) | r.8268_8269ins[8269-6_8269-1] | p.(Lys2756_Val2757insLeuGln) | 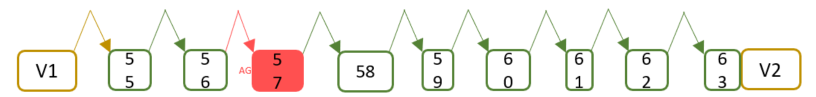 |
| △(E57) | r.8269_8418del | p.(Val2757_Met2806del) | 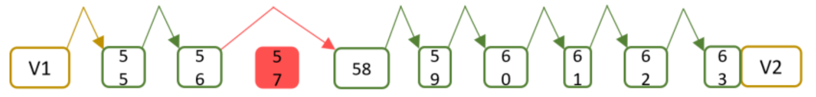 |
| △(E57p18) | r.8269_8286del | p.(Val2757_Gln2762del) | 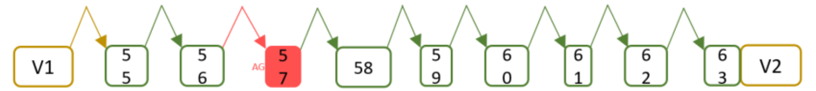 |
| ▼(E57q23a)[1] | r.8418_8419ins[8418+1_8418+4;a;8418+6_8418+23]<br>PTC-NMD | p.(Glu2807Valfs*7) | 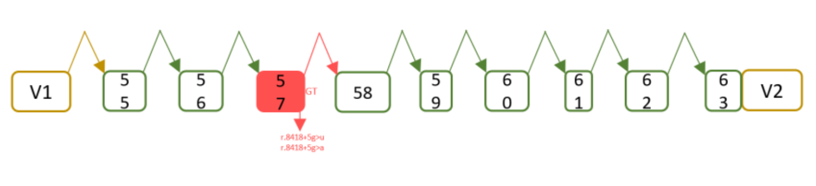 |
| ▼(E57q23b)[1] | r.8418_8419ins[8418+1_8418+4;u;8418+6_8418+23]<br>PTC-NMD | | |
| △(E58) | r.8419_8584del<br>PTC-NMD | p.(Glu2807Leufs*16) | 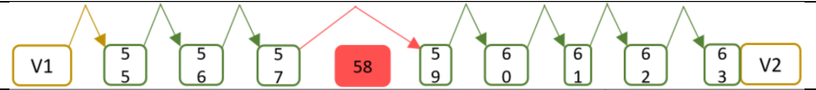 |
| △(E58q50) | r.8535_8584del<br>PTC-NMD | p.(Trp2845Cysfs*9) | 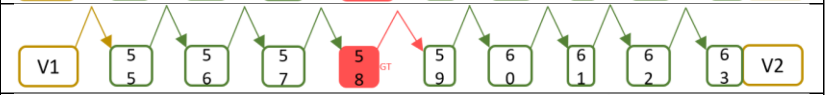 |
| △(E58q19) | r.8566_8584del<br>PTC-NMD | p.(Val2856Leufs*16) | 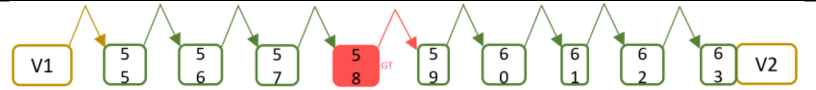 |
| ▼(E58q11) | r.8584_8585ins[8584+1;c;8584+3_8584+11]<br>PTC-NMD | p.(Val2862Glyfs*20) | 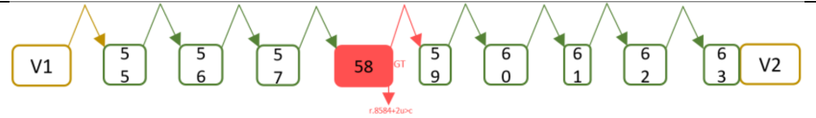 |

| | | | |
|---|---|---|---|
| ▼(E58q48) | r.8584_8585ins[8584+1;c;8584+3_8584+48] PTC-NMD | p.(Val2862Glyfs*10) | 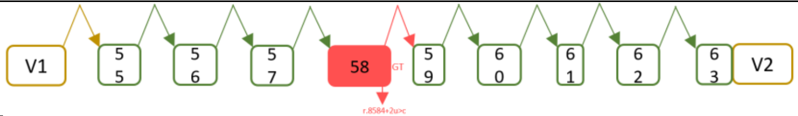  |
| ▼(E58q117a)[1] | r.8584_8585ins[a;8584+2_8584+117] PTC-NMD | p.(Val2862Aspfs*10) | 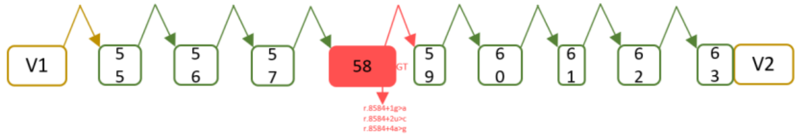  |
| ▼(E58q117b)[1] | r.8584_8585ins[8584+1;c;8584+3_8584+117] PTC-NMD | p.(Val2862Glyfs*10) | |
| ▼(E58q117c)[1] | r.8584_8585ins[8584+1_8584+3;g; 8584+5_8584+117] PTC-NMD | p.(Val2862Glyfs*10) | |
| △(E57_E58) | r.8269_8584del PTC-NMD | p.(Val2757Leufs*16) | 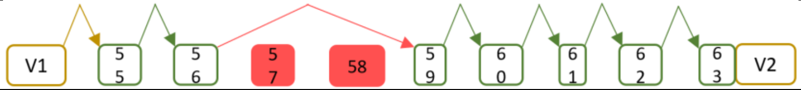  |
| △(E59) | r.8585_8671del | p.(Val2862_Leu2890del) | 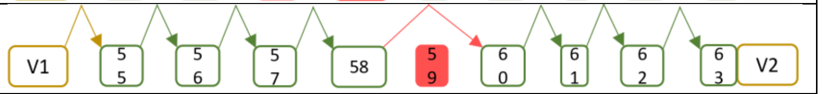  |
| △(E58_E59) | r.8419_8671del PTC-NMD | p.(Glu2807Valfs*47) | 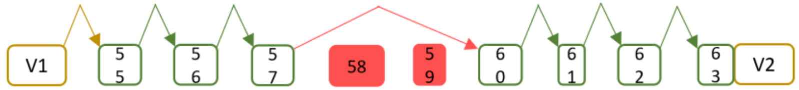  |
| △(E60p17) | r.8672_8688del PTC-NMD | p.(Val2892Glnfs*7) | 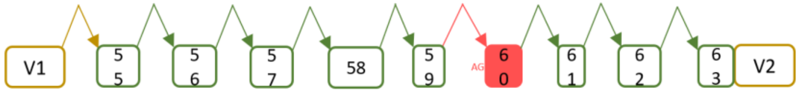  |
| △(E60) | r.8672_8786del PTC-NMD | p.(Gly2891Aspfs*9) | 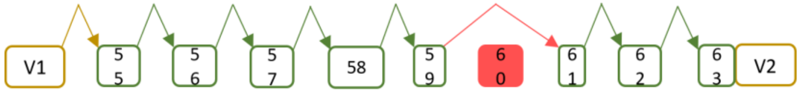  |
| ▼(E60q14) | r.8786_8787ins[a;8786+2_8786+14] PTC-NMD | p.(Cys2930*) | 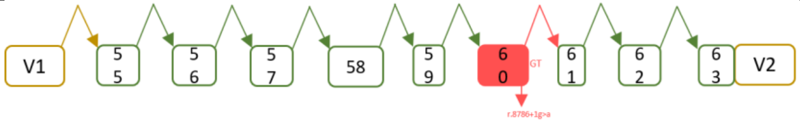  |
| △(E61) | r.8787_8850del PTC-NMD | p.(Cys2930Serfs*12) | 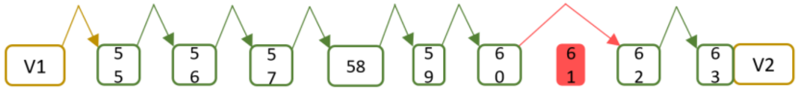  |
| △(E62p49) | r.8851_8899del PTC-NMD | p.(Val2951Leufs*18) | 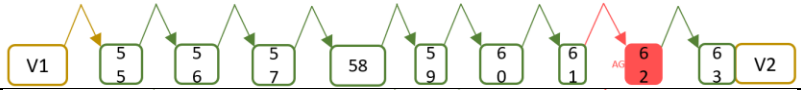  |
| △(E62p63) | r.8851_8913del | p.(Val2951_Gln2971del) | 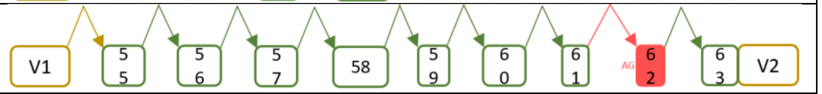  |

| | | | |
|---|---|---|---|
| △(E62p66) | r.8851_8916del | p.(Val2951_Gln2972del) | 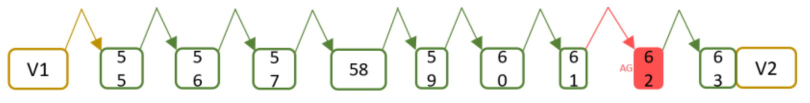 |
| ▼(E62q20) | r.8987_8988ins[8987+1_8987+4;<br>c;8987+6_8987+20]<br>PTC | p.(Ser2996Argfs*2) | 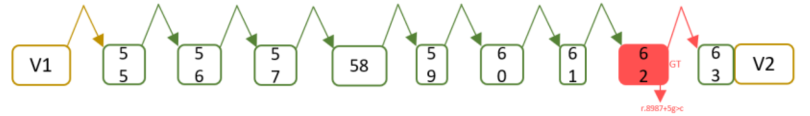 |
| ▼(I62) | r.8987_8988ins<br>PTC | p.(Ser2996Argfs*2) | 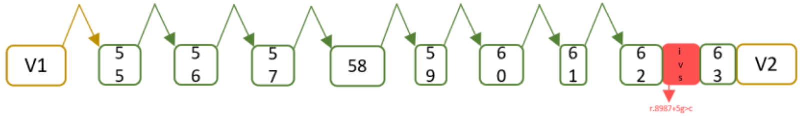 |

[1] The annotations a/b/c reflect the same splicing event with different sequences.
