# Supplementary material for: Experimental Mis-Splicing Assessment and ACMG/AMP-Guided Classification of 47 ATM Splice-Site Variants
Source: Int J Mol Sci. 2026 Jan 12;27(2):765. doi: 10.3390/ijms27020765 (PMC12840730; doi:10.3390/ijms27020765)
Supplement: Supplementary file 1 [file ijms-27-00765-s001.zip › Supplementary_Table_S5_Mutagenesis_Primers_ATM.pdf]

Supplementary Table S5. Cloning and mutagenesis primers for *ATM*.

**Cloning primers**	
**Cloning**	**Primer (5'→3')**
ATM_ex17-18del F	CGAATTGGAGCTCCACCGCGGTGGCGGCCGTTTTGTGTTTTTAGTA
ATM_ex17-18del R	TGAAACTCCATCTCTACTAAAAACACAAAACGGCCGCCACCGCGGT
Overlapping *ATM* ex41-42 F	CGGCCGCTCTAGAACTAGTGGATCCCCCGGTGAACTGTATTTCAGAACTGTATTTCAGAA
Overlapping *ATM* ex41-42 R	TATCGATAAGCTTGATATCGAATTCCTGCATCTCCCTGAATTTTTTCTCTTTTTTTTGCA
Overlapping *ATM* ex43 F	TGCAAAAAAAAGAGAAAAAATTCAGGGAGATGATATTTTGGGATTTTAAATGATATTGTG
Overlapping *ATM* ex43 R	TATCGATAAGCTTGATATCGAATTCCTGCAAAACAACTCCTGTTATATTCATAGAAGAGA
Overlapping *ATM* ex44 F	TCTCTTCTATGAATATAACAGGAGTTGTTTTGAGTAATTTCCTTTTTTTCTGCTTAAAGA
Overlapping *ATM* ex44 R	GACGGTATCGATAAGCTTGATATCGAATTCGTAAAGAATGAACCTGGAATCCCAGAAACA
ATM_55-63V2	GGAAGAAGGCACTGTGCTCAGTGTTGCCTGGAGATCTCCCGAGGGGACCC<br>GGGTCCCCTCGGGAGATCTCCAGGCAACACTGAGCACAGTGCCTTCTTCC

**Variants**	**Primers (5'→3')**
c.2839-2A>T	TTTTTCCCTCCTACCATCTTTGTATCTAATGCTTTTAAAGG<br>CCTTTAAAAGCATTAGATACAAAGATGGTAGGAGGGAAAAA
c.2921C>T<br>p.(Ser974Phe)	TGAACTTCTGAAACCACTATTGTAAGAAATTAAAACCTTAT<br>ATAAGGTTTTAATTTCTTACAATAGTGGTTTCAGAAGTTCA
c.2922-1G>A	TTTTTTTTTTTTTTACCACAACAATGTGTGTTCTTTGTATC<br>GATACAAAGAACACACATTGTTGTGGTAAAAAAAAAAAAAA
c.3077G>A<br>p.(Trp1026*)	TACAGTAATTGGAGCATTTTAGTAGGTACAGTCTATTTTGT<br>ACAAAATAGACTGTACCTACTAAAATGCTCCAATTACTGTA
c.3077+3A>C	AGTAATTGGAGCATTTTGGTCGGTACAGTCTATTTTGTGGT<br>ACCACAAAATAGACTGTACCGACCAAAATGCTCCAATTACT
c.3078-10T>G	TTTAACTTTGGAAAACTTACGTGATTTCAGGCATCTAACAA<br>TTGTTAGATGCCTGAAATCACGTAAGTTTTCCAAAGTTAAA
c.3078-1G>A	GGAAAACTTACTTGATTTCAAGCATCTAACAAAGGAGAGGA<br>TCCTCTCCTTTGTTAGATGCTTGAAATCAAGTAAGTTTTCC
c.3078G>T<br>p.(Trp1026Cys)	GAAAACTTACTTGATTTCAGTCATCTAACAAAGGAGAGGAA<br>TTCCTCTCCTTTGTTAGATGACTGAAATCAAGTAAGTTTTC
c.3153G>T<br>p.(Glu1051Asp)	TGCCTTAAAACTTTGCTTGATGTGAGTTTTTGCATTTTTTT<br>AAAAAAATGCAAAAACTCACATCAAGCAAAGTTTTAAGGCA
c.3153+4A>G	TTAAAACTTTGCTTGAGGTGGGTTTTTGCATTTTTTTAGTA<br>TACTAAAAAAATGCAAAAACCCACCTCAAGCAAAGTTTTAA
c.3154-7C>A	TATTTAACCACAGTTCTTTTACCGTAGGCTGATCCTTATTC<br>GAATAAGGATCAGCCTACGGTAAAAGAACTGTGGTTAAATA
c.3154-6C>T	ATTTAACCACAGTTCTTTTCTCGTAGGCTGATCCTTATTCA<br>TGAATAAGGATCAGCCTACGAGAAAAGAACTGTGGTTAAAT
c.3284G>A<br>p.(Arg1095Lys)	GGCTGCAGAGTCAATCAATAAGTAATGGGTCAAATATTCAT<br>ATGAATATTTGACCCATTACTTATTGATTGACTCTGCAGCC
c.3284G>C<br>p.(Arg1095Thr)	GGCTGCAGAGTCAATCAATACGTAATGGGTCAAATATTCAT<br>ATGAATATTTGACCCATTACGTATTGATTGACTCTGCAGCC
c.3284+1G>A	GCTGCAGAGTCAATCAATAGATAATGGGTCAAATATTCATG<br>CATGAATATTTGACCCATTATCTATTGATTGACTCTGCAGC
c.3284+4A>G	GCAGAGTCAATCAATAGGTAGTGGGTCAAATATTCATGAAG<br>CTTCATGAATATTTGACCCACTACCTATTGATTGACTCTGC
c.6095G>A<br>p.(Arg2032Lys)	GATGTTACAACCCATTACTAAGTAAATTGCATTTTTCTAAA<br>TTTAGAAAAATGCAATTTACTTAGTAATGGGTTGTAACATC
c.6095+4A>G	TTACAACCCATTACTAGGTAGATTGCATTTTTCTAAACAA<br>GTTGTTTAGAAAAATGCAATCTACCTAGTAATGGGTTGTAA
c.6095+6T>C	ACAACCCATTACTAGGTAAACTGCATTTTTCTAAACAACGG<br>CCGTTGTTTAGAAAAATGCAGTTTACCTAGTAATGGGTTGT
c.6096-2A>G	TTTTCACAATCTTTTCTTATGGACTACGAACATATGAACAC<br>GTGTTCATATGTTCGTAGTCCATAAGAAAAGATTGTGAAAA
c.6198+1G>A	GCCAGGCAGGAATCATTCAGATACATTTTTTCCCAGATTTG<br>CAAATCTGGGAAAAAATGTATCTGAATGATTCCTGCCTGGC
c.6348-2A>T	TTTTCTTTGACTTATCTCACTGCAAAGAAGTAGAAGGAACC<br>GGTTCCTTCTACTTCTTTGCAGTGAGATAAGTCAAAGAAAA
c.6348-6_6348-5del	AGTATATTTTTTTCTTTGACTTATCACAGCAAAGAAGTAGAAGGAACCAG<br>CTGGTTCCTTCTACTTCTTTGCTGTGATAAGTCAAAGAAAAAAATATACT
c.6348-10T>A	GTATATTTTTTTCTTTGACTAATCTCACAGCAAAGAAGTAG<br>CTACTTCTTTGCTGTGAGATTAGTCAAAGAAAAAAATATAC
c.6451A>G<br>p.(Arg2151Gly)	ATGAAAGTCTCAAATATGCCGGGTATTATGAAAAGACAAAG<br>CTTTGTCTTTTCATAATACCCGGCATATTTGAGACTTTCAT
c.8011-2A>S	ATCATGTTTATACTTTTATTSGGTGGACCACACAGGAGAAT<br>ATTCTCCTGTGTGGTCCACCSAATAAAAGTATAAACATGAT
c.8152G>T<br>p.(Gly2718Cys)	CATGCTTAATTATTCTGAAGTGCCGTGATGACCTGAGACAA<br>TTGTCTCAGGTCATCACGGCACTTCAGAATAATTAAGCATG

c.8269-7A>G	TAAAAGGTATTTAATCTGTAGCTCCAGGTGGTTCCCCTCTC<br>GAGAGGGGAACCACCTGGAGCTACAGATTAAATACCTTTTA
c.8269-2A>T	GGTATTTAATCTGTAACTCCTGGTGGTTCCCCTCTCTCAGC<br>GCTGAGAGAGGGGAACCACCAGGAGTTACAGATTAAATACC
c.8418+5_8418+8del	CAGTGCCAAAAGAAAATGATGGTGACACCCAAAATTAAAGGTTATTGTAA<br>TTACAATAACCTTTAATTTTGGGTGTCACCATCATTTTCTTTTGGCACTG
c.8418+5G>W	CCAAAAGAAAATGATGGTGAWTGACACCCAAAATTAAAGGT<br>ACCTTTAATTTTGGGTGTCAWTCACCATCATTTTCTTTTGG
c.8584+1G>A	TGTAGCTACTTCTTCTATTGATAATCTTCTTGTACATATAG<br>CTATATGTACAAGAAGATTATCAATAGAAGAAGTAGCTACA
c.8584+2T>C	GTAGCTACTTCTTCTATTGGCAATCTTCTTGTACATATAGT<br>ACTATATGTACAAGAAGATTGCCAATAGAAGAAGTAGCTAC
c.8584+4A>G	AGCTACTTCTTCTATTGGTAGTCTTCTTGTACATATAGTAG<br>CTACTATATGTACAAGAAGACTACCAATAGAAGAAGTAGCT
c.8671+1G>A	ACTTGTACATATAGATCTAGATAAGTAATAAAATCTATGTA<br>TACATAGATTTTATTACTTATCTAGATCTATATGTACAAGT
c.8671+2T>A	CTTGTACATATAGATCTAGGAAAGTAATAAAATCTATGTAT<br>ATACATAGATTTTATTACTTTCCTAGATCTATATGTACAAG
c.8672-3T>G	CTTCACTGTATTCTTTACTTGAGGTGTTGCTTTTGAACAGG<br>CCTGTTCAAAAGCAACACCTCAAGTAAAGAATACAGTGAAG
c.8786+1G>A	GTTGAAGGTGTCTTCAGAAGATAAGTGATATGAAGTAAAGG<br>CCTTTACTTCATATCACTTATCTTCTGAAGACACCTTCAAC
c.8850G>T<br>p.(Glu2950Asp)	ACTCTGTTAACCATTGTAGATGTAAAGTATTTTATAAGGAA<br>TTCCTTATAAAATACTTTACATCTACAATGGTTAACAGAGT
c.8850+4A>C	TGTTAACCATTGTAGAGGTACAGTATTTTATAAGGAAGACT<br>AGTCTTCCTTATAAAATACTGTACCTCTACAATGGTTAACA
c.8851-3T>G	ATACATATGTTCTCTCTGTTGAGGTCCTTCTATATGATCCA<br>TGGATCATATAGAAGGACCTCAACAGAGAGAACATATGTAT
c.8851-1G>Y	ACATATGTTCTCTCTGTTTAYGTCCTTCTATATGATCCACT<br>AGTGGATCATATAGAAGGACRTAAACAGAGAGAACATATGT
c.8987+5G>C	GCAAACGAAATCTCAGGTGACCAGTATTTTAAGAAGGTCCT<br>AGGACCTTCTTAAAATACTGGTCACCTGAGATTTCGTTTGC
c.8988-7_8988-5del	CCTCACTGAAACCTTTGTGTTTTTGTTAGTGATATTGACCAGAGTTTCAA<br>TTGAAACTCTGGTCAATATCACTAACAAAAACACAAAGGTTTCAGTGAGG
